# The Public Health Concerns of Marijuana Legalization: An Overview of Current Trends

**DOI:** 10.7759/cureus.5806

**Published:** 2019-09-30

**Authors:** Valeriy Zvonarev, Tolulope A Fatuki, Polina Tregubenko

**Affiliations:** 1 Psychiatry, School of Behavioral Sciences, California Southern University, Costa Mesa, USA; 2 Psychiatry, Spartan University of Health Sciences, Saint Lucia, USA; 3 Internal Medicine, Jacobi Medical Center - Albert Einstein College of Medicine, New York, USA

**Keywords:** marijuana legalization, cannabis, hospitalization, policy, outcomes, public health, crime rate, addiction

## Abstract

The objective of this study was to conduct a review of the benefits and adverse effects of cannabis (or marijuana) legalization in various states across the US. The current study offers a preliminary evaluation of the problems concerning marijuana legalization in several states, with the primary goal being the assessment of the impact of laws and policies governing the legalization and use of marijuana for medical purposes. A comprehensive search on cannabis and its derivatives was performed using multiple resource databases: PubMed, MEDLINE, Embase, PsycINFO, CENTRAL (Cochrane Controlled Register of Trials), government web sources, and the Department of Public Health databases. A total of 47 reports that evaluated the effects of cannabis legalization were included in this review. All review stages were conducted independently by two reviewers. Data were extracted in standardized tables by one reviewer and adjusted by a second, which were verified by the third author. We examined the use of cannabis before and after the changes in policy and the impact of marijuana legalization on traffic safety, behavior and educational achievement in adolescents, public health, tax revenues, criminal justice expenditures, and financial outcomes. We analyzed the effects and consequences of marijuana use in states that have or have not legalized marijuana. This report also includes the responsiveness of the people in states where marijuana is legalized and its value in the healthcare system. Our study highlights the existing limitations of reviews that probe the effect of decriminalizing marijuana in some states of the country. Our analysis shows that detailed and precise evaluation of policy dynamics must be conducted, taking into account the heterogeneity in population sub-groups and policies. Accordingly, in states where marijuana is used for its medicinal value and recreational purposes, people have different views on the legalization of marijuana. The complete effect of legalizing and commercializing marijuana on consumers' mental health and their educational outcomes is expected to take a longer duration prior to its achievement; unfortunately, fewer merits are anticipated. Most of the reports evaluated in this article proved to be marred with inconsistencies. Many of the stated claims did not pass a methodical evaluation. Going forward, additional data from available sources will lead to stronger conclusions. We weighed the pros and cons of marijuana legalization. However, we are certain that consumers can make better decisions by weighing each opinion by its reliability and safety.

## Introduction and background

The process of marijuana decriminalization started in 1970 with medical professionals having access to it for medical purposes. In the United States eight states and Washington, D.C. voted to legalize adult use of marijuana. Marijuana was first legalized by the state legislature in Vermont in January 2018. Marijuana use is legal in Colorado, Alaska, California, Montana, Oregon, Vermont, Hawaii, Nevada, Washington, and Maine for recreational and medical purposes. Advocates believe that legalization raises revenue, lowers criminal justice expenditure, improves public health and traffic safety, and stimulates the economy. Furthermore, legalization aimed to reduce the crime rate reported in these states. Critics believe legalization exacerbates marijuana use, increases crime and raises legal issues, affects public health and safety, and lowers teen educational achievement. There is no clear answer to whether legalizing marijuana can save lives and protect our public health system.

We identified primary studies, systematic reviews, official reports, and state-issued documents addressing the effects of marijuana legalization in various states. We also assessed the medical use of marijuana in different “marijuana-unfriendly” jurisdictions in the US. Our main hypothesis was that the legalization of marijuana is associated with various negative outcomes. 

We established criteria that provided the basis for making decisions consistent with desired outcomes and research goals.

Each state was analyzed using the following criteria: 

1. Marijuana use before and after legalization

2. Marijuana use in youth

3. Violent crime rate

4. Fatal car crashes and accidents

5. Admissions to substance abuse treatment facilities 

6. Drug-related emergency department (ED) visits 

7. Alcohol- and drug-induced death and suicide rates 

8. Marijuana revenues

The trends and patterns are expected to vary before and after legalization in the states included in this article. Thus, the available information provides a useful perspective on what other states should expect from legalization and the related policies. 

The legalization of marijuana in the US has received several opinions on the changes in the rate of marijuana use as well as other factors associated with its consumption. Our analysis includes Alaska, California, Colorado, Maine, Massachusetts, Nevada, Oregon, Washington, and other states as well as Washington, D.C. A summary of the marijuana legalization timeline is presented in Table 1. 

**Table 1:** Marijuana legalization and the start of adult-use retail sales by state Source: [1]

Ballot Measure	Date Ballot Measure Passed	Date Possession Legalized	Date Retail Sales Began	Number of Non-Medical Stores in Operation as of 11/7/2016
Alaska	11/4/2014	2/24/2015	10/1/2016	147
California	11/8/2016	11/9/2016	State-level retail licenses are expected to start being issued on 1/1/2018; issuance of local licenses will vary by locality.	None
Colorado	11/6/2012	12/10/2012	1/1/2014	504
Maine	11/8/2016	1/30/2017	Retail licenses are expected to be issued on 2/1/2018	None
Massachusetts	11/8/2016	12/15/2016	Retail sales are expected to commence on 7/18/2018	None
Nevada	11/8/2016	1/1/2017	Sales through existing medical marijuana outlets started on 7/1/2017	37
Oregon	11/4/2014	7/1/2015	Early retail sales of marijuana to adults 21 and over began at medical marijuana dispensaries on 10/1/2015 and licenses for adult-use retailers began issuing on 10/2/2016.	507
Washington	11/6/2012	12/6/2012	7/8/2014	516
Washington, D.C.	11/4/2014	2/26/2015	Retail sales remain unlawful	None

Colorado legalized marijuana in 2012, with changes in trends before and after the legalization period. Table 1 shows that California legalized marijuana on November 9, 2016 following a vote cast on November 8, 2016. Retail sales of marijuana commenced on January 1, 2018 after the provision of licenses by the state. 

The state of Washington legalized marijuana on December 6, 2012, on recreation grounds, thereby becoming one of the states permitting the use and sale of this drug. The proposal on permissive use was launched in 1998, before authorization in 2012 for medical and recreational use among adults of age >21 years. The state of Oregon legalized marijuana on July 1, 2015, with the retail sales commencing immediately. The move was taken since the inception of the ballot on November 8, 2014. This formed the earliest legalization period on marijuana within Oregon with 147 non-medical retail stores established to realize marijuana sales after authorization.

In Vermont, 2011-2012 had the highest consumption of marijuana at 13.4% as compared to 7.5% in the entire country. The levels dropped to 11.3%, 11.4%, and 10.9% in the preceding years. 

In Michigan, the vote for marijuana use was proposed in 2008 and later legalized in November 2018. The impact of legalization changed the use of cannabis, thereby becoming the first illicit substance legalized within the state of Michigan. 

**Figure 1:**
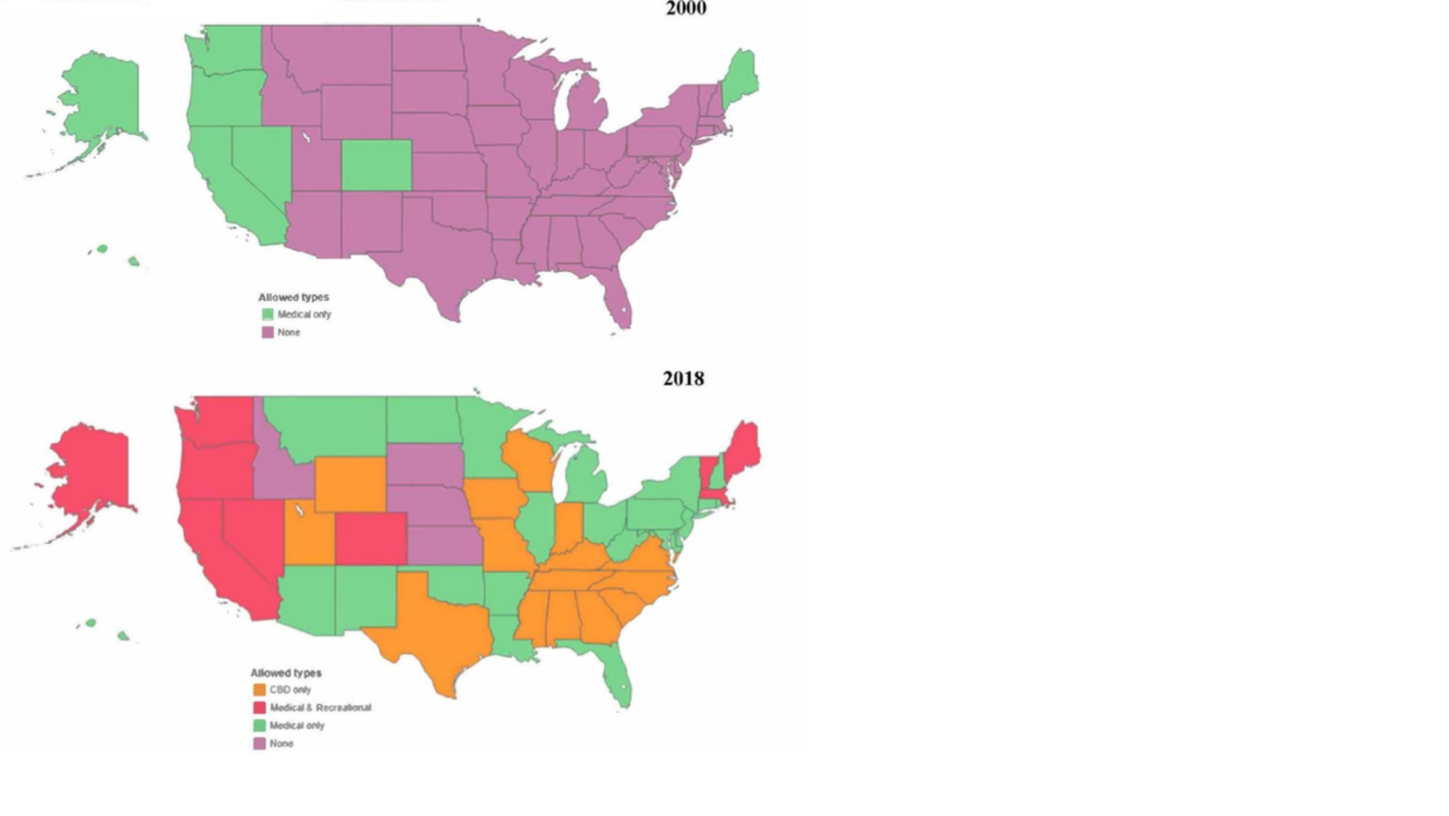
State marijuana legalization status, 2018 Source: National Conference of State Legislatures. http://www.ncsl.org/research/health/state-medical-marijuana-laws.aspx CBD, cannabidiol

Figure 1 represents the legalization status of marijuana across the US, for medical or recreational purposes, as detailed in Table 1. The maps also illustrate the expansion of marijuana to several states post-2012.

## Review

Part 1: Cannabis use before and after legalization**

The effect of marijuana legalization for recreational purposes on the rate of marijuana use has been a topic of considerable debate. Table 2 presents the percentage of past-year marijuana use among persons aged ≥12 years, categorized by age group (2002-2014).

**Table 2:** Percentage of the past-year marijuana use* among all persons aged ≥12 years, by age group / per state — National Survey on Drug Use and Health Source: Substance Abuse and Mental Health Services Administration, Center for Behavioral Health Statistics and Quality, National Survey on Drug Use and Health, 2002–2014 CI, confidence interval *Past-year marijuana use is defined as those who reported the use of marijuana within 12 months preceding the date of interview, which also included those who reported 30 days preceding the date of interview.

Colorado	2002-2003	2003-2004	2004-2005	2005-2006	2006-2007	2007-2008	2008-2009	2009-2010	2010-2011	2011-2012	2012-2013	2013-2014
	% (95% CI)	% (95% CI)	% (95% CI)	% (95% CI)	% (95% CI)	% (95% CI)	% (95% CI)	% (95% CI)	% (95% CI)	% (95% CI)	% (95% CI)	% (95% CI)
Age group (years)												
Total	15.7 (13.8-17.7)	14.1 (11.9-16.6)	12.0 (10.0-14.2)	12.5 (10.6-14.7)	13.7 (11.8-15.9)	14.3 (11.9-17.1)	15.7 (12.9-19.0)	18.5 (15.2-22.3)	16.7 (14.6-18.9)	15.9 (13.2-19.1)	19.2 (15.7-23.3)	20.7 (17.9-23.8)
12–17	19.1 (15.9-22.9)	18.8 (15.5-22.6)	17.3 (14.3-20.9)	16.8 (13.7-20.4)	17.7 (14.1-22.0)	17.4 (13.7-21.9)	20.0 (16.1-24.5)	20.5 (16.9-24.7)	20.0 (16.5-24.1)	17.1 (13.9-21.0)	18.4 (14.6-23.0)	22.8 (18.8-27.3)
18–25	35.9 (30.9-41.2)	30.2 (25.8-35.0)	32.2 (26.7-38.4)	36.8 (30.9-43.1)	35.3 (30.5-40.4)	35.4 (29.7-41.5)	37.4 (29.5-45.9)	39.9 (32.7-47.5)	42.7 (37.6-48.1)	41.6 (36.0-47.5)	44.2 (38.2-50.3)	45.2 (40.0-50.6)
≥26	11.6 (9.5-14.2)	10.6 (8.0-14.0)	7.6 (5.6-10.3)	7.7 (5.9-10.0)	9.5 (7.4-12.2)	10.4 (7.9-13.7)	11.6 (8.8-15.1)	14.5 (11.1-18.8)	11.7 (9.7-14.1)	11.4 (8.5-15.1)	15.1 (11.2-19.9)	16.3 (13.2-19.9)

California	2002-2003	2003-2004	2004-2005	2005-2006	2006-2007	2007-2008	2008-2009	2009-2010	2010-2011	2011-2012	2012-2013	2013-2014
	% (95% CI)	% (95% CI)	% (95% CI)	% (95% CI)	% (95% CI)	% (95% CI)	% (95% CI)	% (95% CI)	% (95% CI)	% (95% CI)	% (95% CI)	% (95% CI)
Age group (years)												
Total	11.7 (10.6-13.0)	11.4 (10.3-12.6)	11.8 (10.8-12.9)	11.8 (10.8-12.9)	11.6 (10.5-12.7)	11.8 (10.6-13.0)	13.0 (11.9-14.2)	13.8 (12.6-15.1)	14.1 (12.9-15.4)	14.1 (13.0-15.3)	14.1 (12.9-15.5)	14.7 (13.6-15.9)
12–17	13.6 (12.3-15.1)	14.5 (13.1-15.9)	13.8 (12.4-15.4)	12.9 (11.5-14.5)	12.9 (11.5-14.5)	13.1 (11.7-14.8)	14.9 (13.3-16.8)	15.8 (14.0-17.9)	17.0 (15.1-19.1)	16.0 (14.4-17.8)	13.6 (12.1-15.3)	14.6 (13.0-16.5)
18–25	28.5 (26.2-30.9)	27.7 (25.2-30.4)	27.7 (25.5-30.0)	28.7 (26.4-31.1)	28.2 (26.0-30.5)	29.4 (27.1-31.8)	31.7 (29.3-34.2)	32.4 (30.0-34.9)	32.5 (30.3-34.8)	33.6 (31.0-36.3)	34.8 (32.1-37.6)	34.3 (31.9-36.9)
≥26	8.4 (7.2-9.8)	8.0 (6.8-9.4)	8.6 (7.5-10.0)	8.6 (7.4-9.9)	8.2 (7.1-9.6)	8.2 (6.9-9.7)	9.1 (7.9-10.6)	10.0 (8.6-11.5)	10.3 (8.9-11.9)	10.2 (9.0-11.7)	10.4 (9.0-11.9)	11.2 (9.9-12.5

Alaska	2002-2003	2003-2004	2004-2005	2005-2006	2006-2007	2007-2008	2008-2009	2009-2010	2010-2011	2011-2012	2012-2013	2013-2014
	% (95% CI)	% (95% CI)	% (95% CI)	% (95% CI)	% (95% CI)	% (95% CI)	% (95% CI)	% (95% CI)	% (95% CI)	% (95% CI)	% (95% CI)	% (95% CI)
Age group (years)												
Total	16.2 (14.3-18.2)	16.2 (14.3-18.3)	17.6 (14.8-20.9)	15.6 (12.9-18.7)	14.8 (12.3-17.7)	15.9 (13.4-18.6)	17.2 (14.5-20.3)	18.8 (15.3-22.8)	18.6 (15.9-21.6)	20.6 (18.1-23.2)	20.8 (18.1-23.8)	20.0 (17.3-23.0)
12–17	18.3 (15.1-22.1)	17.6 (14.1-21.9)	16.8 (13.3-21.0)	16.2 (12.9-20.2)	15.6 (12.5-19.4)	15.6 (12.3-19.6)	16.1 (12.8-20.1)	16.5 (13.1-20.7)	17.0 (13.7-20.9)	14.6 (11.7-18.1)	13.9 (11.2-17.1)	17.2 (13.8-21.3)
18–25	40.1 (34.9-45.6)	37.4 (32.7-42.3)	35.1 (30.2-40.3)	33.7 (28.8-39.0)	33.4 (27.5-39.9)	32.3 (26.5-38.8)	34.5 (29.7-39.6)	35.8 (30.3-41.8)	38.4 (32.7-44.4)	39.8 (34.2-45.8)	36.1 (31.2-41.2)	36.6 (31.1-42.6)
≥26	11.7 (9.3-14.5)	11.9 (9.8-14.5)	14.4 (11.0-18.5)	11.9 (8.9-15.9)	11.2 (8.1-15.2)	12.8 (10.0-16.2)	14.1 (10.9-18.1)	15.8 (11.6-21.1)	14.9 (12.1-18.3)	17.8 (15.0-20.9)	18.9 (15.5-22.8)	17.2 (13.9-21

Maine	2002-2003	2003-2004	2004-2005	2005-2006	2006-2007	2007-2008	2008-2009	2009-2010	2010-2011	2011-2012	2012-2013	2013-2014
	% (95% CI)	% (95% CI)	% (95% CI)	% (95% CI)	% (95% CI)	% (95% CI)	% (95% CI)	% (95% CI)	% (95% CI)	% (95% CI)	% (95% CI)	% (95% CI)
Age group (years)												
Total	12.9 (10.7-15.4)	12.6 (10.5-15.1)	12.5 (10.8-14.4)	12.7 (10.5-15.3)	12.3 (10.3-14.5)	12.4 (10.4-14.8)	14.4 (12.1-16.9)	15.0 (13.0-17.3)	14.6 (12.4-17.0)	15.4 (12.9-18.3)	17.1 (14.2-20.4)	20.3 (17.5-23.5)
12–17	18.9 (15.7-22.6)	18.7 (15.3-22.7)	20.1 (15.9-25.0)	18.7 (14.9-23.2)	17.0 (13.7-20.8)	15.8 (13.2-18.7)	12.5 (9.8-15.9)	13.9 (11.0-17.4)	14.7 (11.7-18.3)	15.4 (12.3-19.1)	15.5 (12.5-19.0)	15.9 (12.8-19.5)
18–25	37.9 (32.7-43.3)	38.4 (33.3-43.9)	37.9 (33.4-42.7)	41.5 (37.1-46.0)	41.2 (36.2-46.3)	37.0 (32.6-41.6)	41.6 (35.5-47.9)	44.1 (37.9-50.6)	38.2 (33.4-43.4)	35.8 (32.2-39.4)	39.9 (35.8-44.3)	43.4 (38.5-48.5)
≥26	8.3 (6.3-11.0)	7.9 (5.9-10.5)	7.6 (5.8-9.9)	7.6 (5.4-10.7)	7.6 (5.5-10.3)	8.6 (6.6-11.3)	10.8 (8.7-13.4)	11.0 (8.8-13.6)	11.2 (8.5-14.5)	12.6 (9.7-16.2)	14.1 (11.0-17.9)	17.6 (14.4-21.4)

Massachusetts	2002-2003	2003-2004	2004-2005	2005-2006	2006-2007	2007-2008	2008-2009	2009-2010	2010-2011	2011-2012	2012-2013	2013-2014
	% (95% CI)	% (95% CI)	% (95% CI)	% (95% CI)	% (95% CI)	% (95% CI)	% (95% CI)	% (95% CI)	% (95% CI)	% (95% CI)	% (95% CI)	% (95% CI)
Age group (years)												
Total	16.1 (13.9-18.7)	14.2 (12.0-16.8)	11.7 (9.9-13.8)	12.4 (10.5-14.7)	13.0 (10.7-15.7)	11.3 (9.5-13.4)	14.6 (12.4-17.3)	16.6 (14.2-19.2)	15.2 (13.0-17.6)	15.1 (12.7-17.8)	15.4 (13.0-18.1)	17.1 (14.5-20.1)
12–17	21.3 (17.6-25.5)	18.8 (15.7-22.2)	17.4 (14.0-21.4)	17.4 (13.7-21.7)	17.3 (13.4-22.2)	16.8 (13.5-20.7)	17.1 (14.5-19.9)	17.6 (14.3-21.6)	19.4 (16.4-22.8)	18.9 (15.9-22.4)	14.8 (12.0-18.0)	15.7 (12.8-19.1)
18–25	40.2 (34.9-45.7)	40.4 (35.9-45.1)	38.8 (34.1-43.7)	39.0 (35.4-42.6)	39.0 (34.1-44.1)	36.1 (31.1-41.5)	40.4 (35.0-45.9)	44.9 (39.1-50.8)	43.1 (37.9-48.6)	40.8 (35.0-46.8)	43.5 (38.1-49.0)	44.8 (40.1-49.6)
≥26	11.7 (8.7-15.4)	9.4 (6.9-12.7)	6.7 (4.8-9.1)	7.4 (5.5-10.1)	8.0 (5.9-10.9)	6.3 (4.7-8.6)	9.8 (7.5-12.6)	11.5 (9.0-14.5)	9.8 (7.5-12.6)	10.1 (7.6-13.4)	10.5 (8.3-13.3)	12.5 (9.7-15.8)

Nevada	2002-2003	2003-2004	2004-2005	2005-2006	2006-2007	2007-2008	2008-2009	2009-2010	2010-2011	2011-2012	2012-2013	2013-2014
	% (95% CI)	% (95% CI)	% (95% CI)	% (95% CI)	% (95% CI)	% (95% CI)	% (95% CI)	% (95% CI)	% (95% CI)	% (95% CI)	% (95% CI)	% (95% CI)
Age group (years)												
Total	12.4 (10.5-14.5)	10.7 (9.0-12.7)	10.4 (8.6-12.5)	11.8 (9.7-14.2)	11.9 (9.7-14.6)	11.7 (9.4-14.5)	11.7 (9.3-14.5)	12.4 (9.9-15.2)	13.7 (11.6-16.0)	14.4 (11.8-17.4)	14.3 (11.8-17.2)	12.9 (10.8-15.4)
12–17	20.7 (16.5-25.6)	17.6 (14.6-21.0)	14.4 (11.5-17.9)	14.2 (10.6-18.9)	14.0 (11.4-17.2)	16.4 (13.4-20.0)	17.9 (14.4-22.1)	13.9 (10.2-18.7)	16.3 (11.8-22.1)	18.6 (14.2-24.1)	16.6 (13.2-20.8)	14.9 (11.5-19.1)
18–25	26.9 (23.0-31.3)	24.3 (20.6-28.5)	27.0 (22.2-32.6)	25.6 (21.0-30.8)	22.3 (18.7-26.5)	25.3 (21.0-30.2)	27.1 (22.3-32.4)	27.0 (22.1-32.5)	31.7 (23.0-41.9)	32.0 (23.5-42.0)	29.9 (24.2-36.3)	31.8 (26.8-37.2)
≥26	9.0 (7.0-11.5)	7.6 (5.9-9.9)	7.2 (5.3-9.6)	9.2 (7.0-12.1)	10.1 (7.3-13.7)	9.1 (6.3-12.8)	8.6 (6.0-12.1)	9.9 (7.4-13.3)	10.4 (8.1-13.3)	11.0 (8.0-15.0)	11.5 (8.7-15.1)	9.7 (7.3-12.8)

Oregon	2002-2003	2003-2004	2004-2005	2005-2006	2006-2007	2007-2008	2008-2009	2009-2010	2010-2011	2011-2012	2012-2013	2013-2014
	% (95% CI)	% (95% CI)	% (95% CI)	% (95% CI)	% (95% CI)	% (95% CI)	% (95% CI)	% (95% CI)	% (95% CI)	% (95% CI)	% (95% CI)	% (95% CI)
Age group (years)												
Total	14.8 (11.8-18.4)	13.6 (10.7-17.1)	14.1 (12.1-16.5)	14.0 (12.2-16.2)	14.5 (11.9-17.4)	15.1 (12.6-18.1)	15.4 (13.4-17.7)	16.5 (14.3-18.9)	16.9 (14.7-19.3)	18.4 (15.8-21.4)	20.6 (17.2-24.5)	20.1 (17.3-23.1)
12–17	16.4 (13.3-20.1)	18.1 (14.8-22.0)	19.4 (15.1-24.5)	19.7 (15.3-25.1)	16.5 (13.5-20.1)	16.7 (13.0-21.1)	19.4 (16.0-23.4)	21.0 (17.2-25.4)	18.6 (15.0-22.9)	15.4 (12.6-18.8)	16.4 (13.3-20.1)	18.3 (14.7-22.7)
18–25	36.6 (30.0-43.7)	34.6 (29.3-40.4)	35.9 (31.3-40.8)	32.9 (28.1-38.1)	33.9 (28.5-39.8)	37.7 (33.3-42.3)	38.7 (34.4-43.1)	39.9 (35.4-44.6)	40.4 (35.5-45.4)	38.4 (33.2-43.8)	34.9 (29.5-40.7)	37.7 (32.9-42.8)
≥26	10.9 (7.9-14.8)	9.4 (6.6-13.2)	9.7 (7.5-12.6)	10.2 (8.1-12.8)	11.1 (8.5-14.4)	11.4 (8.9-14.6)	11.4 (9.3-13.9)	12.3 (9.8-15.2)	12.9 (10.4-15.9)	15.6 (12.6-19.2)	18.9 (14.9-23.6)	17.5 (14.4-21.1)

Washington	2002-2003	2003-2004	2004-2005	2005-2006	2006-2007	2007-2008	2008-2009	2009-2010	2010-2011	2011-2012	2012-2013	2013-2014
	% (95% CI)	% (95% CI)	% (95% CI)	% (95% CI)	% (95% CI)	% (95% CI)	% (95% CI)	% (95% CI)	% (95% CI)	% (95% CI)	% (95% CI)	% (95% CI)
Age group (years)												
Total	13.6 (11.2-16.5)	11.9 (9.9-14.2)	11.7 (9.8-13.8)	12.7 (10.4-15.5)	12.5 (10.0-15.4)	13.3 (10.6-16.4)	13.3 (10.9-16.2)	14.3 (11.8-17.3)	15.6 (12.9-18.7)	16.2 (13.5-19.3)	18.5 (15.7-21.6)	19.4 (16.9-22.3)
12–17	17.4 (14.0-21.3)	15.2 (12.0-19.0)	11.4 (8.7-14.8)	13.9 (11.0-17.5)	13.7 (11.4-16.3)	13.5 (10.9-16.6)	15.2 (12.3-18.5)	14.4 (11.8-17.4)	16.9 (13.5-21.0)	16.0 (12.7-20.0)	15.5 (12.4-19.2)	17.4 (13.8-21.7)
18–25	36.5 (31.8-41.6)	28.7 (23.3-34.8)	29.5 (24.3-35.4)	32.7 (27.2-38.6)	31.4 (26.1-37.2)	29.8 (25.4-34.5)	33.8 (29.6-38.4)	39.9 (34.4-45.7)	40.5 (34.6-46.7)	37.2 (32.4-42.3)	37.9 (33.0-43.1)	34.3 (29.2-39.8)
≥26	9.2 (6.8-12.3)	8.5 (6.2-11.5)	8.6 (6.6-11.1)	9.2 (6.9-12.2)	9.2 (6.6-12.6)	10.6 (7.7-14.4)	9.8 (7.0-13.5)	10.1 (7.4-13.7)	11.3 (8.6-14.7)	12.7 (9.7-16.5)	15.7 (12.4-19.6)	17.3 (14.2-20.8)

District of Columbia	2002-2003	2003-2004	2004-2005	2005-2006	2006-2007	2007-2008	2008-2009	2009-2010	2010-2011	2011-2012	2012-2013	2013-2014
	% (95% CI)	% (95% CI)	% (95% CI)	% (95% CI)	% (95% CI)	% (95% CI)	% (95% CI)	% (95% CI)	% (95% CI)	% (95% CI)	% (95% CI)	% (95% CI)
Age group (years)												
Total	15.3 (12.9-18.0)	14.4 (12.1-17.0)	15.2 (12.9-17.8)	14.8 (12.7-17.1)	15.9 (13.5-18.6)	15.9 (13.7-18.3)	14.4 (11.7-17.6)	16.3 (13.2-19.9)	18.7 (15.4-22.5)	20.3 (17.3-23.7)	20.8 (17.7-24.4)	21.4 (18.9-24.1)
12–17	15.1 (12.3-18.4)	12.1 (9.7-15.1)	14.5 (11.3-18.4)	13.5 (10.7-17.0)	12.9 (10.1-16.2)	14.6 (11.9-17.8)	16.8 (13.0-21.6)	19.0 (14.9-24.0)	18.8 (15.1-23.1)	17.8 (15.1-20.9)	19.5 (16.2-23.4)	21.7 (18.1-25.7)
18–25	37.2 (32.7-41.9)	30.3 (25.4-35.8)	32.8 (28.0-38.1)	37.5 (32.8-42.6)	39.0 (33.6-44.6)	37.0 (32.0-42.4)	32.8 (28.6-37.2)	33.5 (29.9-37.4)	37.5 (33.0-42.2)	42.8 (37.6-48.2)	42.6 (37.1-48.3)	38.9 (34.1-44.0)
≥26	11.0 (8.3-14.3)	11.5 (9.2-14.4)	12.1 (9.6-15.1)	10.5 (8.5-13.0)	11.2 (8.6-14.3)	11.3 (9.0-14.1)	10.0 (7.2-13.8)	12.2 (8.9-16.4)	14.4 (10.8-19.0)	15.2 (11.9-19.2)	16.1 (12.6-20.2)	17.6 (14.6-20.9)

Table 3 presents the annual average percentages of marijuana use in the past year, categorized by age group and state (2016 and 2017). This data shows the pre- and post-legalization rates of marijuana use. 

**Table 3:** Marijuana use in the past year, by age group and state: Percentages, annual averages based on 2016 and 2017 NSDUHs Note: State and census region estimates, along with the 95% Bayesian confidence (credible) intervals, are based on a survey-weighted hierarchical Bayes estimation approach and generated by Markov Chain Monte Carlo techniques. For the "Total US" row, design-based (direct) estimates and corresponding 95% confidence intervals are given. Source: Substance Abuse and Mental Health Services Administration, Center for Behavioral Health Statistics and Quality, National Survey on Drug Use and Health, 2016 and 2017 NSDUH, National Survey on Drug Use and Health

State	12+ (Estimate)	12+ (95% Confidence Interval)						12-17 (Estimate)	12-17 (95% Confidence Interval)			18-25 (Estimate)		18-25 (95% Confidence Interval)				26+ (Estimate)	26+ (95% Confidence Interval)		18+ (Estimate)	18+ (95% Confidence Interval)	
Total US	14.50	(14.20-14.80)						12.19	(11.77-12.63)			33.91		(33.17-34.66)				11.61	(11.27-11.95)		14.73	(14.41-15.06)	
Northeast	15.10	(14.51-15.71)						12.29	(11.48-13.15)			37.92		(36.47-39.39)				11.77	(11.06-12.51)		15.36	(14.73-16.02)	
Midwest	13.88	(13.37-14.41)						12.74	(12.04-13.47)			33.63		(32.50-34.78)				10.74	(10.12-11.39)		14.00	(13.44-14.57)	
South	12.25	(11.82-12.69)						10.99	(10.38-11.62)			30.28		(29.30-31.29)				9.49	(8.96-10.05)		12.38	(11.91-12.86)	
West	18.17	(17.51-18.85)						13.58	(12.69-14.51)			36.86		(35.44-38.29)				15.62	(14.82-16.45)		18.64	(17.93-19.38)	
Alaska	22.73	(20.53-25.10)						16.51	(13.99-19.39)			39.44		(35.37-43.66)				20.74	(18.20-23.53)		23.43	(21.06-25.98)	
California	17.39	(16.46-18.37)						13.30	(11.95-14.79)			36.53		(34.37-38.74)				14.63	(13.54-15.80)		17.81	(16.79-18.87)	
Colorado	24.86	(22.61-27.25)						16.97	(14.25-20.08)			48.81		(44.51-53.12)				21.88	(19.31-24.69)		25.65	(23.24-28.23)	
District of Columbia	26.35	(24.04-28.81)						14.60	(12.07-17.56)			53.20		(48.47-57.87)				21.90	(19.34-24.69)		27.01	(24.59-29.57)	
Maine	21.94	(19.74-24.31)						15.81	(13.26-18.75)			45.67		(41.47-49.92)				19.36	(16.85-22.13)		22.46	(20.11-24.99)	
Massachusetts	19.96	(17.96-22.12)						16.39	(13.77-19.39)			44.93		(40.59-49.36)				16.03	(13.83-18.51)		20.28	(18.16-22.58)	
Nevada	16.82		(14.82-19.02)					14.37	(11.94-17.19)				35.81	(31.75-40.08)			14.34			(12.11-16.90)	17.06		(14.93-19.43)
Oregon	26.51		(24.19-28.97)					17.01	(14.37-20.01)				47.57	(43.39-51.78)			24.36			(21.70-27.22)	27.38		(24.89-30.02)
Washington	22.49		(20.26-24.89)					14.97	(12.51- 17.81)				42.12	(37.97-46.39)			20.30			(17.78-23.07)	23.21		(20.81-25.78)

Marijuana use has been on a constant rise in the US, from 2002 to 2017, as indicated in the above tables. The increase in usage has been recognized among young people aged ≥12 years.

1. Alaska

In Alaska there has been a fluctuating increase in the number of marijuana users, probably because during this period (2002-2017) marijuana still remained illegal in most states of the country. As of 2014, the total percentage of marijuana users stood at 20.0%. The state has experienced fluctuations in the number of users over this period. On February 24, 2015, the possession of marijuana was legalized in the state. This legalization and the emergence of retail traders for marijuana resulted in an increase in the number of users (24.5%) in the state.

2. California

The state of California experienced a continuous rise in the percentage of marijuana users from 2002 (11.7%) to 2014 (14.7%), indicating a gradual increase of 3% over the years. On September 11, 2016 the state legalized marijuana possession, but no trade retailers were authorized yet. This, in turn, resulted in an increase of up to 19.93% across the state.

3. Colorado

In 2002 the percentage of marijuana users in this state was 15.7%, a figure that rose steadily up to 20.7% in 2014, indicating a 5% increase over the years. The state of Colorado was among the first states to legalize the possession of marijuana on June 11, 2012. Nearly 504 retailers of the drug appeared in the state, thus increasing the number of users to 27.7% by the end of 2017. This increase demonstrates a direct association between the legalization of the drug and the number of users in the state.

4. Maine

The state of Maine had registered quite a significant percentage in the number of marijuana users from 2002 (12.9%) to 2014 (20.3%). These figures were quite surprising primarily because the state took longer to legalize the use of marijuana. The possession of the drug was made legal in the state on January 30, 2017. This legalization translated to a further increase in the number of drug users in the state, recording 25.04% by the end of 2017.

5. Massachusetts

The state of Massachusetts recorded the smallest increase in the number of marijuana users between 2002 and 2014 (16.1% to 17.1%). The drug was legalized on December 15, 2016. There was a significant increase after the legalization of the drug in the state, with the percentage being 21.88% by the end of 2017.

6. Nevada

In 2002 the percentage of people using marijuana was 12.1% and 12.9% by the end of 2014. This was quite low as compared to most of the other states in the country. On January 1, 2017 Nevada legalized the possession of marijuana, which saw the percentage of users rise to 19.6% by the end of the year.

7. Oregon

The state of Oregon had 14.8% marijuana users in 2002 and 20.1% in 2014. The percentage of marijuana users gradually increased over the years, which further increased after the state legalized the drug on July 1, 2015, up to 28.16% by the end of 2017.

8. Washington

The state of Washington had 13.6% marijuana users, which increased to 19.4% by the end of 2014. The percentages rose further after the legalization of the drug in the state on June 12, 2012, with the figures being 24.61% by the end of 2017.

According to the data presented, the legalization of the possession of marijuana in the sampled states led to an increase in the number of users.

Part 2: Cannabis use in youth 

Several states analyzed in this report have legalized the use of marijuana for adults. However, according to our analysis, the rate of marijuana consumption among the youth has remained stable with a decreasing rate in some states, which was against the expectations of many. Interestingly, the rate of marijuana consumption has drastically reduced among younger teenagers, while the number of older teenagers consuming marijuana remains stable, with the primary reason being the fact that marijuana is not as easily accessible to the young teens as it had been in previous years [1-2]. 

Eight states and Washington, D.C. have legalized the possession of limited amounts of marijuana for adult use. It should be noted that these policies are in different stages of implementation. Presently, Colorado, Washington, Alaska, Oregon, and, to a lesser extent, Nevada and California have implemented state-level policies and retail marijuana sales; up-to-date state-based youth use data are not yet available for Oregon, Nevada, or California. 

Youth marijuana use has remained relatively stable in the past several years, both nationwide and in states with established marijuana regulatory programs [1]. According to the 2015 Youth Risk Behavior Survey, 21.7% of the American high school students used marijuana in the past month, and this rate has been consistent over the past decade. Rates of marijuana use by high school students in Washington, Colorado, and Alaska largely resemble these national rates, with only a few variations. These results are promising, suggesting that the fear of a widespread increase in use has not come to fruition.

**Figure 2:**
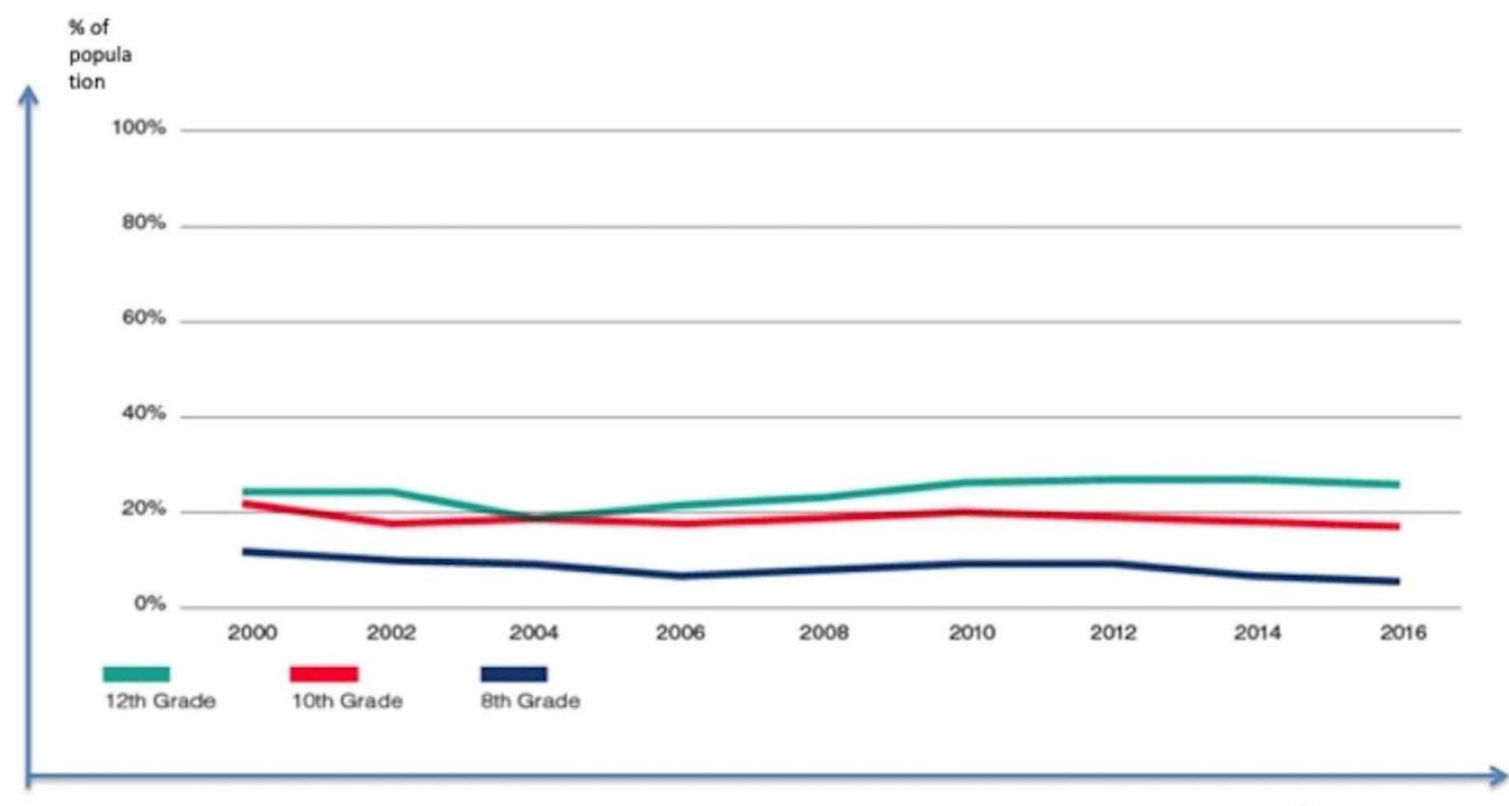
Past 30-day marijuana use in Washington by grade Source: [1]

The Washington State Healthy Youth Survey results for marijuana use by 8th, 10th, and 12th graders from 2000 to 2016 suggest that use rates have either remained the same or decreased. The results indicate that the past 30-day use of marijuana by 10th and 12th graders in the state has remained statistically unchanged for the past several years, with legalization having little or no impact on these rates [3]. The rates of use by 10th and 12th graders in Washington, 17% and 26%, respectively, remain similar to or slightly lower than the national rates for these grades, which are 20% and 27.6%, respectively. Recent use among 8th graders has fallen to 6%, a 50% decline since 2000. The consumption rate of marijuana among youth has not been influenced greatly by the regulations set by a given date, as these regulations are not yet fully implemented or they are so new that they are unlikely to have an impact on marijuana use among youth. The rate of marijuana use, although varied widely in these states, mostly stabilized or declined over the years leading up to legalization. Figure 2 shows cannabis use in Washington by grade. 

Before legalization, the rate of marijuana use by youth in Oregon, Massachusetts, and Washington, D.C. did not match the nationwide trend with higher and lower rates demonstrated by Massachusetts, Washington, D.C., and Oregon, respectively. Consequently, change in legislation cannot be considered as a crucial factor influencing the level of marijuana consumption by the youth. So, as compared to the nationwide average, Washington, D.C. reported higher rates (32.2%) of past 30-day marijuana use by high school students in 2015. These numbers have been high for a while [1]. The lowest rates of use among the surveyed students were registered in Oregon in 2015, where the figures were as low as 8.8% for the eighth graders and 19.1% for the 11th graders. Overall, marijuana use by minors has increased in the states of Colorado and Washington for the past 10 years, according to the survey by the National Survey on Drug Use and Health (NSDUH). Since 2011, the 9th and 10th graders in Colorado have demonstrated virtually stable rates of use. The figures in 2015 were less than half the rate in 2001, due to the number of reported cases of recent use by the 9th graders. Although Colorado saw a rise in recent use rates among 11th and 12th graders, the numbers did not exceed the maximum rates before the legalization of marijuana [1]. YRBS conducted in Alaska in 2003-2017 showed that since 2010, the past 30-day rate of marijuana use has remained stable at 21.5% among high school students and is in line with high school rates nationwide [1]. Of the most recent states to legalize marijuana, teen marijuana use rates appear to be consistent with national averages in California, Nevada, and Maine. By contrast, these rates have remained higher in Massachusetts for some time.

**Figure 3:**
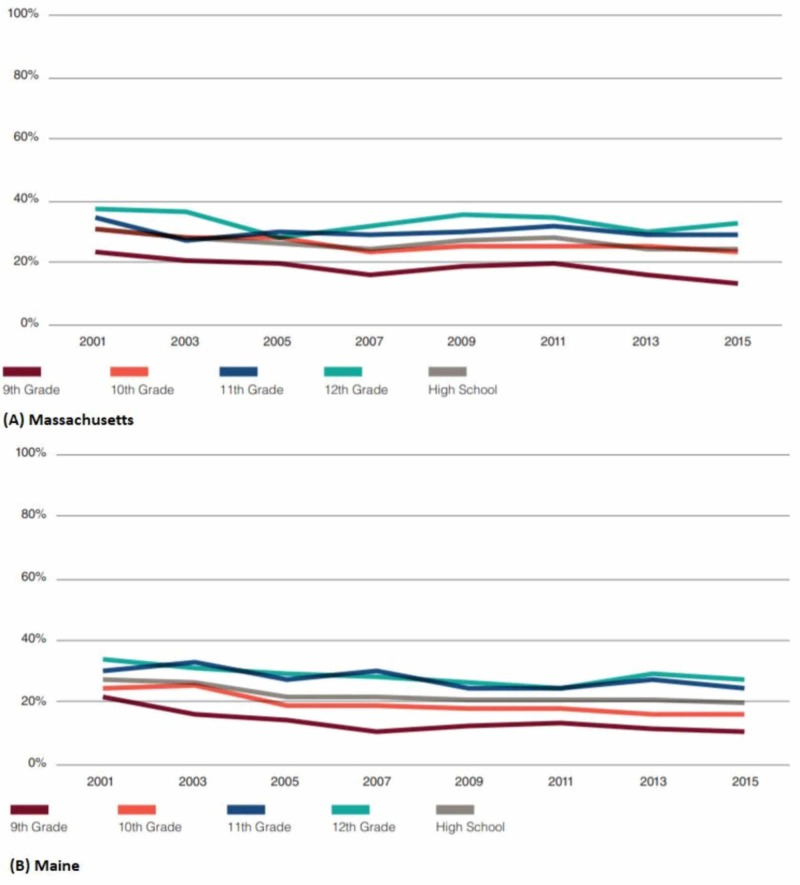
Past 30-day marijuana use in (A) Massachusetts and (B) Maine by grade Source: [1] Teen marijuana use rates appear to be consistent with national averages in Maine. However, they are higher in Massachusetts.

Nevada, for example, demonstrated a one-third decrease in the number of 11th graders in the 2001 to 2015 period (from 30.8 to 21.8). In fact, the same period saw an over 25% reduction in high school rates due to the decrease in marijuana use by 9th graders from 21.6% to 14.6%, 10th graders from 21.8% to 17.8%, and 12th graders from 33.5% to 24.3% [4]. In 2015, the high schools of Maine registered half as many 9th graders who reportedly used marijuana within 30 days as compared to 2001. Similarly, compared to 2001, other grades demonstrated decreasing rates of 20% to 25% [4]. Besides, the marijuana use rate in high schools in Massachusetts was less in 2015 (24.5%) compared to 2001 when 30.9% of the students reported using marijuana within 30 days [4]. California, however, demonstrated stable figures for marijuana use by the 9th and 12th graders since 2004 [5]. Figure 3 shows the past 30-day marijuana use in Maine and Massachusetts by grade.

Marijuana arrests involving underage youth have reduced due to marijuana legalization. Nevertheless, the youth may contribute to a large number of marijuana arrests. For instance, in Oregon, marijuana arrests of underage youth declined by 80%, while for adults, the arrest rates reduced by 92% between 2015 and 2012 [1]. Nevertheless, in 2016 underage youths arrested for marijuana offenses totaled approximately seven times that of adults. Likewise, in Washington convictions of marijuana possession reduced by 99.1% for the adults, while there was a 56% decline in youth convicts between 2015 and 2012. In 2015, underage youths represented 98% of all marijuana-related convictions. Decriminalizing marijuana and reducing penalties for underage youth would help reduce marijuana arrests. For example, California has abolished all incarcerations and financial penalties for underage youth and introduced a requirement for any youth indulging in marijuana consumption in the state to be educated about drug awareness and to seek counseling and participate in community service. California teens will thus be protected from the impact of felony charges [1]. 

Part 3: Violent crime due to marijuana use 

In 2010 legalization supporters stated that the legalization of cannabis would eliminate the underground market for marijuana and reduce violent crime associated with the illegal sale of this illicit substance. No changes were observed in crime after medical marijuana commercialization, legalization adoption, or full legalization implementation. The increase in homicides is associated with marijuana legalization and credited mainly to a jump in violent crime in pro-marijuana jurisdictions [1]. In Denver, the homicide rate has steadily climbed to a peak of 67 cases in 2018.

According to the report by the Drug Enforcement Administration, the state of Colorado has experienced a worrying upsurge of criminal networks since 2014. For example, the Mayor of Colorado Springs stated that “Mexican cartels are no longer sending marijuana into Colorado, they’re now growing it in Colorado and sending it back to Mexico and every place else.” The U.S. Postal Service has recorded an escalating amount of black-market Colorado marijuana bound to other jurisdictions. 

Similarly, in Seattle, there was an increase from 19% to 31% in 2018 over the preceding year. According to Seattle Police Department statistics, it is evident that by the end of 2018, violent crime had increased by 9% and property crime had risen by 2%, with a 2% overall increase in all crime [6]. The total shots fired decreased from 360 in 2017 to 312 in 2018. By contrast, hate crimes increased from 113 in 2017 to 125 in 2018. There is little room for error since the material has the correct official crime data recorded in Seattle. 

Homicide cases have also increased in the District of Colombia, from 116 in 2017 to 160 in 2018 [7]. It could be concluded that the legalization of marijuana affects crime rates. The demand for marijuana increased as users are less afraid of arrest, coupled with subsidized or low-cost marijuana. 

According to the San Francisco sheriff's office, a production facility with the capacity to process more than 500 million pounds of marijuana was raided and seized. Shockingly, only 20% of the marijuana grown in California is legal; the rest is illegal and trafficked to other states. The high demand for marijuana, coupled with novel and advanced growing techniques, has contributed to the production of half a million pounds of illegal marijuana. Illegally grown marijuana rose from 700,000 pounds in 2017 to 1.6 million pounds in 2018 despite legalization. Moreover, the shutdown of the illicit cannabis business seems to be ineffective as new businesses are soon reaping the benefits. Licensed cannabis dealers are complaining of cannibalizing their revenues as they have to pay taxes that illicit companies do not. However, various credible sources and data conclude that the illegal marketing of marijuana is doing better post-legalization [8].

Table 4 summarizes the murder rates in major US cities before and after legalization and suggests the increase in violent crime rates, especially homicides.

**Table 4:** Murder rates in major US cities before and after cannabis legalization Source: American Violence; FBI Uniform Crime Reports FBI, Federal Bureau of Investigation

City and State	Year of Legalization	Murder Rates before Marijuana Legalization (2010; rate per 100k)	Murder Rates after Marijuana Legalization (2018; rate per 100k)	Murder Rates in All Cities >250,000 (2010; Rate per 100k)	Murder Rates in All Cities >250,000 (2017; rate per 100k) * 2018 data is not yet available
Denver, CO	2012	4.7	7.8	10,0	11.0
Seattle, WA	2012	2.6	4.3	10,0	11.0
Washington, D.C.	2015	20.8	22.7	10,0	11.0
San Francisco, CA	2016	6.0	4.7	10,0	11.0
Anchorage, AK	2015	4.5	9.1 (2017)	10,0	11.0
Boston, MA	2016	10.3	7.9	10,0	11.0
Augusta, ME	2017	information is not available	information is not available	10,0	11.0
Las Vegas, NV	2017	18.3	21.1	10,0	11.0
Portland, OR	2015	3.8	4.0	10,0	11.0

Some of the largest cities in the US saw an increase in the homicide rates from 2010 to 2018. The recent increase in violent crime and homicides in large cities has received a great deal of attention, but the role of cannabis still remains unclear. The “Ferguson effect” is one of the more widely discussed and controversial explanations for the recent increase in violent crime. It refers to the assertion that crime has increased in the past several years because police are avoiding proactive policing tactics toward cannabis users due to fear of repercussions for the use of aggressive tactics. However, our analysis did not reveal a causative association between the decreasing arrests, marijuana legalization, and increasing crime.

Marijuana arrests have declined dramatically over the years in states that have legalized marijuana. Moreover, the corresponding revenue has been allocated to support social services such as education and health programs. 

Arrests in all marijuana-friendly jurisdictions and Washington, D.C. for the possession, cultivation, and sale of marijuana have plummeted since voters legalized the adult use of marijuana, saving those states millions of dollars and preventing the criminalization of thousands of young Americans.

Marijuana arrests in Washington, D.C. and all states where marijuana has been legalized have plummeted, as Alaska saw a 93% decline in marijuana arrests due to possessions and sales between 2013 and 2015. In Colorado, there was a 49% decline in arrests for marijuana offenses between 2012 and 2013. However, there was a 7% increase in marijuana arrests in 2014. Court filings due to marijuana offenses in Colorado decreased by 81% between 2012 and 2015. In Oregon, marijuana arrests reduced by 96% between 2013 and 2016. In Washington, D.C., there was a 76% decrease in marijuana arrests between 2013 and 2016. Moreover, the number of people convicted of marijuana possession decreased by 76% between 2011 and 2015. Possession arrests in Washington, D.C. reduced by 98.6% between 2013 and 2016.

Part 4: Fatal car crashes and accidents: association with marijuana legalization

Road Safety

It is unlawful to drive under the influence of marijuana in every state in the country. The National Highway Transport Safety Administration has conducted research, which shows that unlike alcohol, there is no clear correlation between the specific levels of Δ-9-tetrahydrocannabinol (THC) and road safety.

THC can be detected in the bloodstream even after several days of consuming marijuana. This limitation causes unjust punishments and is said to be very unscientific. These tests tend to be unspecific and unpredicted. Alternatives to the THC blood test, which are effect-based, have been put in place. Effect-based tests, such as the Standard Field Sobriety Tests (“SFSTs”) that rely on the observations of trained drug recognition experts (DRE), offer an alternative to the THC threshold tests. At present, the SFSTs have been validated for the identification of alcohol use-associated impairment, but their sensitivity to marijuana impairment is not well established [1]. 

Driving Under the Influence Arrests

**Figure 4:**
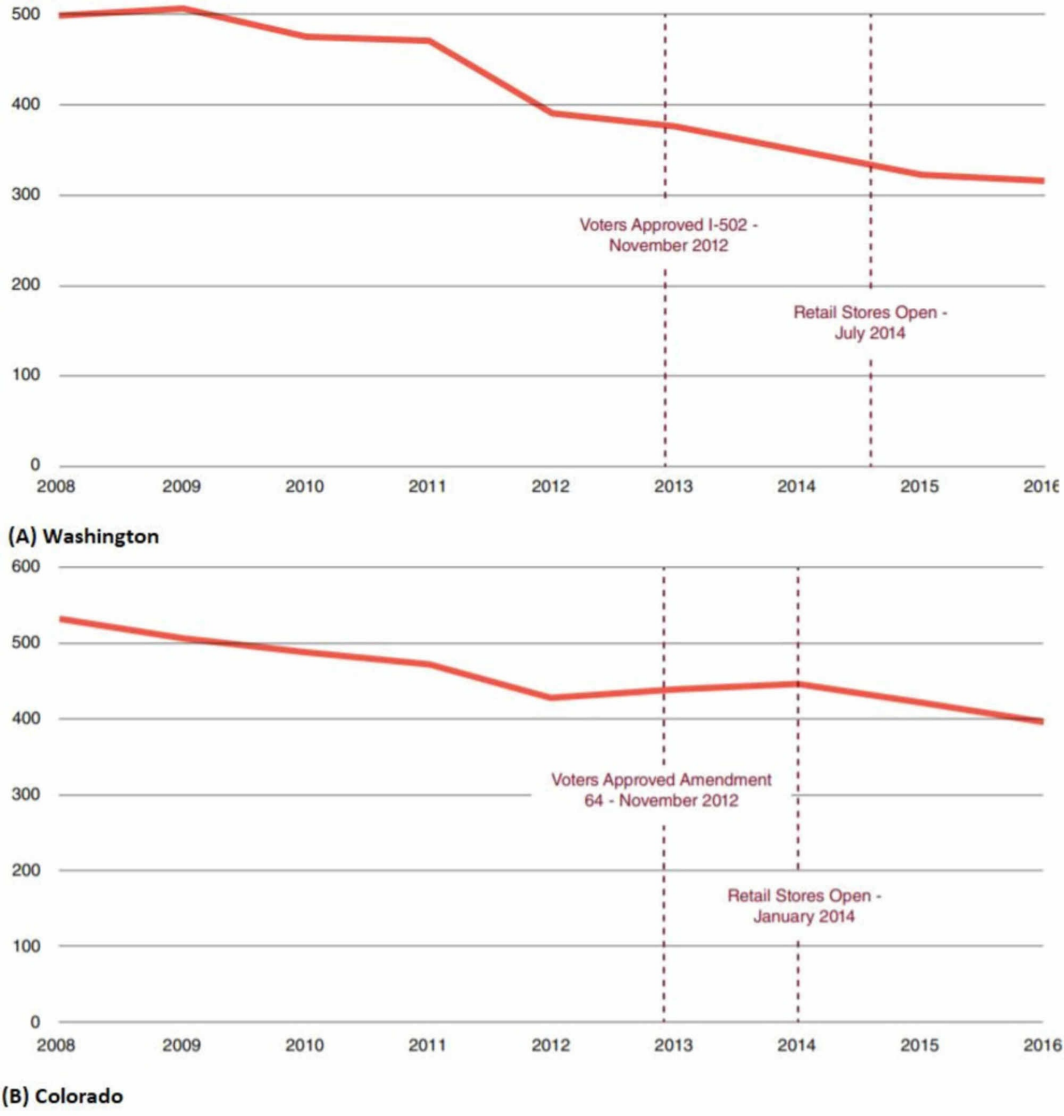
DUI arrest rates in (A) Washington and (B) Colorado per 100,000 people Source: [1] DUI, driving under the influence

In general, the number of individuals arrested under the influence of marijuana has decreased over time in all cannabis-friendly states in the country. In fact, the figures have reduced after the legalization of marijuana in all states. Results from research show that the process of legalization of marijuana does not tend to increase the driving under the influence (DUI) rates in any state. The Colorado Bureau of Investigation reported a 16% state-wide drop in the number of DUI tickets dispensed, from 26,146 in 2011, the year before legalization, to 21,953 in 2016, the second year after legal sales of adult-use marijuana began [1]. Washington saw a 32.9% decline in the number of arrests for any DUI from 34,256 in 2011, the year before marijuana legalization, to 22,993 in 2016, two years following the start of legal sales of marijuana for adult use [1]. However, according to the Washington Traffic Safety Commission, the percentage of DUI cases related to driving while intoxicated has risen considerably in Washington since legalization. The DUI arrest rates in Washington and Colorado have been presented in Figure 4. 

Available evidence confirms an insignificant percentage of DUI arrests in Washington (8%) and Colorado (4%) involving drivers who tested positive for THC or THC metabolites only [1]. In the next three jurisdictions that legalized marijuana for adult use in 2014, namely Oregon, Alaska, and D.C., the data for the periods after legalization or several years prior to legalization are absent or not sufficient. Table 5 provides additional data about the same.

**Table 5:** DUI arrests in Oregon, Washington, D.C., and Alaska Source: [1] DUI, driving under the influence

States	DUI arrests
Oregon	DUI arrests and citations decreased by 25% from 17,341 in 2013 (last year prior to marijuana legalization) to 11,882 in 2015, (the first year after legalization)
Washington, D.C.	DUI arrests declined by 18.3% (1,648 cases in 2013 vs. 1,346 in 2015)
Alaska	Arrests started to increase in 2015 (the first year after legalization, 3,161 cases) but declined again in 2016 due to new retail stores opened (3,063 cases)

Crash Rates and Risk Assessment

There is no clear or stated correlation between the legalization of marijuana and crash rates. The number of drivers who tested positive for THC was significant. While there was an uptick in the number of drivers involved in fatal accidents who tested positive for THC in Washington and Colorado in 2015, there is no evidence between this increase and driver impairment [9]. 

Marijuana was the most common substance detected among drivers who tested positive for drugs (45% overall) and has been the most prevalent drug among positive drug drivers in California since 2007. There was a 36% increase over the 10-year period in the presence of cannabis among drivers who tested positive for drugs, from 37% to 50%.

The test only shows that the drivers used marijuana hours or days before the crash. This could imply an increased use of marijuana among adults following legalization. These results show that there is a change in how THC analysis and data reporting procedures are done after the legitimization of marijuana: for example, before legalization, both states did not routinely test drivers to determine whether THC was involved in a fatal crash. 

**Figure 5:**
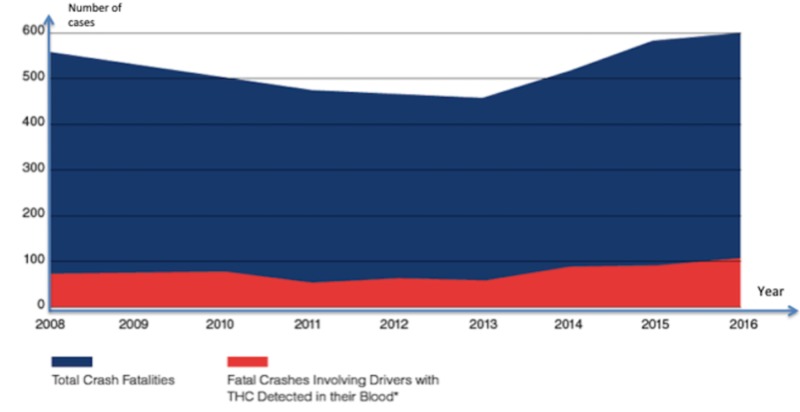
Crash fatalities in Washington *Fatal crashes involving drivers with THC detected in their blood indicate that the drivers tested positive for consumption of THC sometimes in the weeks preceding the crash. Source: Fatality Analysis Reporting System (FARS), WTSC Serious Injury Data Source: Collision Location Analysis System (CLAS), Washington State Department of Transportation (WSDOT) THC, tetrahydrocannabinol

According to the results of the research, fatal crash rates in Colorado and Washington after legalization are not associated with marijuana. Further, the crash rates in the states mentioned above were comparable to the states that have not legalized the use of marijuana [10]. Figure 5 shows crash fatalities in Washington with additional detail. 

There are no clear findings on how the use of marijuana impairs driving, and this topic is debatable. The correlation between alcohol and crash risk is clear, and the findings are consistent. Some studies show that THC only causes minimal impairment with little contribution to crash risk. Some researchers concluded that the use of marijuana alone does not cause any impairment [1]. Other findings also show that occasional marijuana users get impaired while heavy consumers do not [11]. All these results are contradictory and inconclusive about the effects of THC toxicity and driving impairment.

The Department of Transport and National Traffic Safety Administration (USA) conducted research and concluded that no correlation exists between the THC levels in the participant’s blood and impaired driving [1]. Research conducted at the University of Heidelberg showed that THC levels could be detected in a patient even after several days of consumption of marijuana. Medical marijuana patients also had no signs of impairment [12]. These conclusions further supported that the marijuana-related effect is ambiguous and that further research needs to be carried out to establish the association between marijuana and driving impairment. 

Part 5: Admissions to substance abuse treatment facilities 

**Figure 6:**
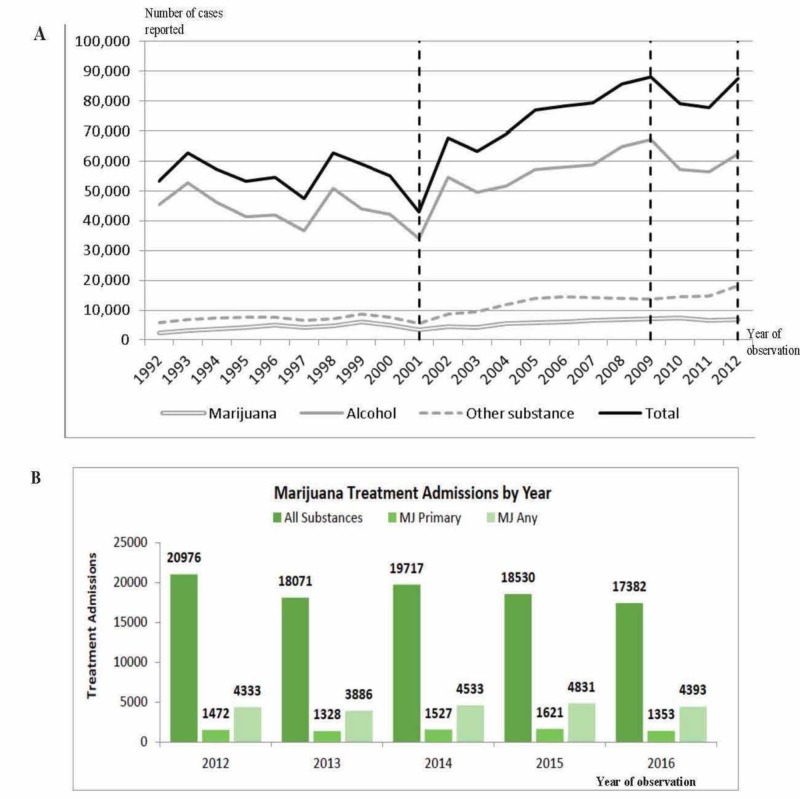
Admissions to substance abuse treatment facilities in Colorado Source: A: Center for Behavioral Health Statistics and Quality, SAMHSA, Treatment Episode Data Set – Admissions (TEDS-A) Series, Concatenated, 1992-2012,   http://www.icpsr.umich.edu/icpsrweb/SAMHDA/sda B: Trends in Marijuana Treatment Admissions, 2012-2016, Denver, Colorado. Report prepared by Emery Shekiro SAMHSA, Substance Abuse and Mental Health Services Administration; MJ, marijuana

Marijuana use showed a slight increase before 2010 until medical marijuana became readily available in dispensaries, following which marijuana use leveled up. Since legalization, marijuana has experienced a declining pattern in the US, as demonstrated in the analyzed reports. There are concerns that the legalization of marijuana is prone to an increase in health-related expenses, despite the drug being used for medicinal purposes. It was confirmed that more patients present in the ED with physical or psychological symptoms associated with overdose of cannabis products, but no significant changes in the trend have been observed after marijuana introduction. This indicates that alcohol abuse was the highest in terms of the patients admitted to the addiction treatment facilities as compared to marijuana in Colorado. Figure 6 describes admissions to substance abuse treatment facilities in Colorado between 1992 and 2012. Moreover, it also summarizes all treatment admissions, marijuana treatment admissions, and treatment admissions where marijuana was recorded as the primary, secondary, or tertiary substance. Data were collected on all admissions aged ≥12 years. 

After the legalization of marijuana, there were no notable fluctuations in the figures of marijuana treatment admissions. More than 20% of the treatment admissions reported the use of marijuana between 2012 and 2016 in cases of admissions related to marijuana-related treatment, with marijuana not being a prime substance. People receiving treatment frequently used marijuana, even if the cause for admission was different.

**Figure 7:**
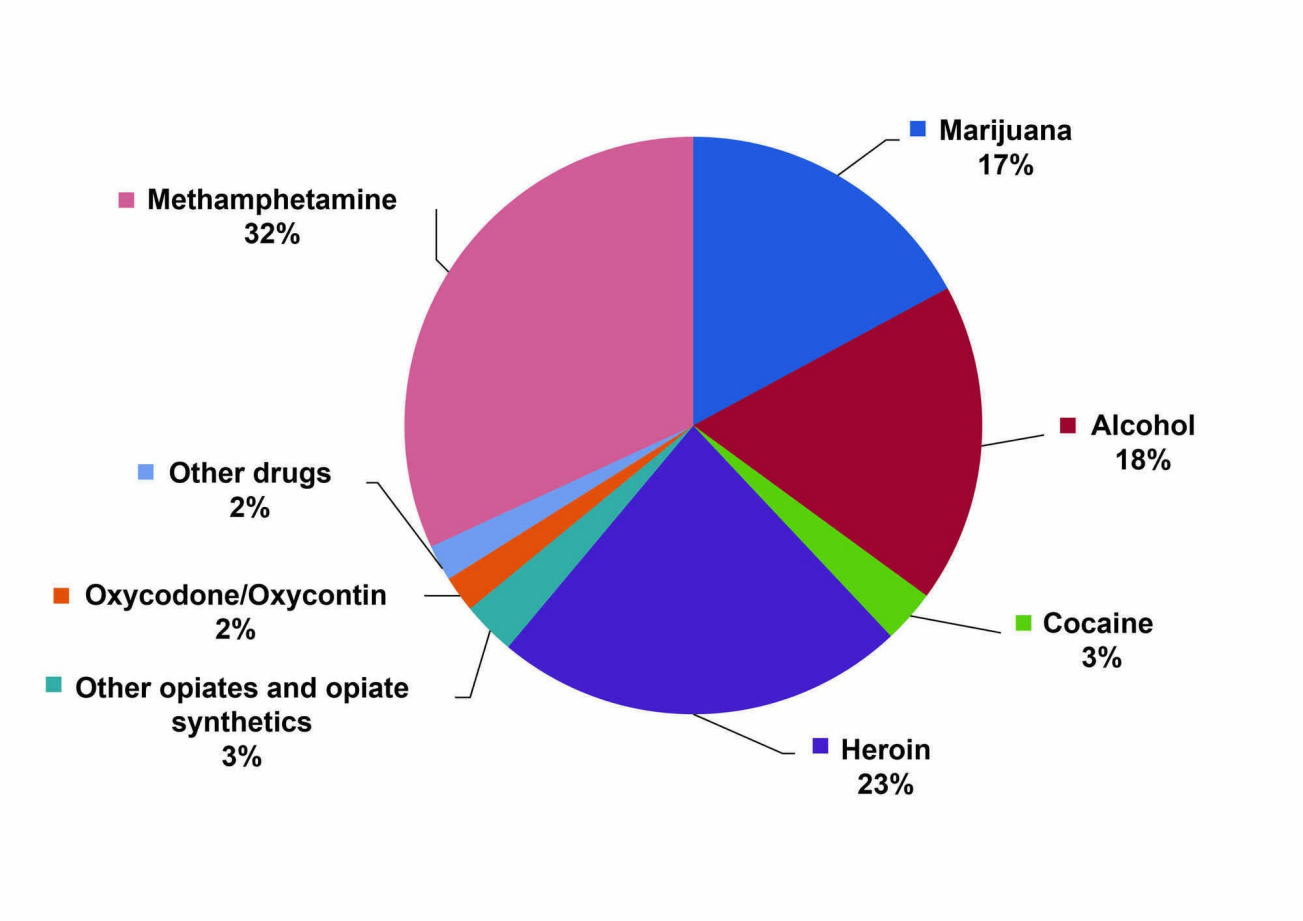
Primary drugs reported at admission to publicly funded substance use disorder treatment programs Source: California Outcomes Measurement System Treatment (CalOMS Tx) data, Department of Health Care Services (Reported in 2017 Statewide Needs Assessment and Planning Report). 2014-2015. http://publichealth.lacounty.gov/sapc/prevention/cannabis/LTCChapter3DataandInformation.pdf

Cannabis is the third most prevalent primary drug for treatment (17%), following treatment for methamphetamine (32%) and heroin (23%), in California. Figure 7 shows the primary drugs reported by beneficiaries of substance use disorder treatment at the time of treatment admission in 2014-2015. 

According to the statewide database that provides data on all beneficiaries receiving substance use disorder treatment services from publicly monitored treatment programs, a significant increase in the number of admissions due to cannabis was observed over a period of 10 years. 

Based on several sources, marijuana was the most commonly cited drug among primary drug treatment admissions in Alaska, followed by opiates and heroin, in 2010. In the 2010 National Survey of Substance Abuse Treatment Services (N-SSATS), Alaska showed a total of 3,218 clients in addiction treatment, the majority of whom (2,835 or 88.1%) underwent outpatient addiction treatment. Of the total number of clients undergoing treatment on this date, 243 were of age <18 years. Unfortunately, Alaska does not have recent data available [13]. Calls to poison control centers in the state of Washington surged 68% from 2012 (pre-legalization) to 2015 and 109% in Colorado over the same timeframe. In this regard, calls in Colorado related to children aged 0 to 8 years rose over 200%. Similarly, hospitalizations related to marijuana in Colorado have increased over 70% since legalization, an average of over 30% per year. The data showing the number of individuals admitted to the rehab facilities in the rest of the states have been summarized in Table 6. 

**Table 6:** Data showing the number of individuals, on average, admitted into the rehab facilities before and after the legalization of marijuana in the states of Washington, D.C., Washington, Nevada, Oregon, Massachusetts, and Maine Data retrieved from TEDS TEDS, treatment episode data set

State	Year of Legalization	Treatment Admissions Before Legalization (Rate per 100k)	Treatment Admissions After Legalization (Rate per 100k)
Washington, D.C.	2015	819 in 2012, 607 in 2014	1,226 in 2016
Washington	2012	6,871 in 2004, 7,705 in 2007, 7,914 in 2012	8,430 in 2013
Nevada	2017	1,461 in 2004, 1,502 in 2007, 1,086 in 2014	1,629 in 2018
Oregon	2015	6,893 in 2004, 7,660 in 2007, 3,664 in 2014	5,120 in 2016
Massachusetts	2016	3,144 in 2004, 4,360 in 2007, 2,652 in 2014	3,105 in 2017
Maine	2017	1,761 in 2004, 1,449 in 2007, 1,196 in 2014	1,789 in 2018

The above data are averaged, implying that they include admissions from all age groups for generalized reasons. However, breaking down the data yields different figures. Admissions are for individuals aged ≥12 years. Note that each state has its pattern of admission rates when taking 2004, 2007, and 2014 as the base years of admissions before legalization and a year after as the base year following legalization. In D.C., there are no stored data of admissions before 2012. Admission rates reduced from 2012 to 2014, and after legalization, admission rates hiked to 1226 in 2014. 

In Washington, the drug was legalized in 2012, and hence, the 2014 data are missing. Admission rates have been increasing significantly in the state and have continued to increase even post-legalization. In Nevada, rates have been varying over the years but increased upon legalization. The same happened in the state of Oregon as well. 

In Massachusetts, the number of admissions has been rising significantly. A survey conducted from 2006 to 2015 concluded that the number of drug-related admissions has been rising significantly due to disorderly use with high rates of admission witnessed after legalization. Most of the hospitalized are teenagers aged 12 to 19 years, who constitute 40% of total admissions. This shows that marijuana legalization has increased consumption in this state [14]. In Maine, rates of admission have been reducing over the years but increased upon legalization.

The overall conclusion that can be drawn from the above data is that state marijuana legalization has had a significant effect on drug consumption rates. The marked feature of this data is that admission rates increased after the legalization of the drug. These can be associated with the removal of drug regulations prohibiting the use of the drug. As the legalization of the drug removes criminal charges related to use and possession, the drug is readily available in the stalls; thus, the increased consumption rates are reflected in the increased number of admission rates. 

Part 6: Drug-related ED visits 

After marijuana legalization, calls to poison control centers for marijuana exposure remain unchanged compared to calls about exposure to other, more common household products and substances. While marijuana-related calls to poison centers in states that legalized marijuana are higher now compared to the pre-legalization years, the number of calls related to marijuana makes up only a small fraction of the total calls and is dramatically lower than the number of calls for items such as prescription drugs. In Oregon, for example, less than 1% of the calls to the state’s poison centers in 2016 were related to marijuana exposure. The state of Washington experienced an increase in calls to its poison center post-legalization for marijuana exposures. Yet, these marijuana-related calls only accounted for 286 of 62,502 calls in 2016. About 75% of individuals calling the Washington poison center for marijuana-related exposures had their cases managed at home, meaning they did not require in-person medical interventions at urgent care centers, EDs, or doctors’ offices.

As a result, poison centers and EDs in states that legalized marijuana experienced, as expected, an increase in calls and visits for adverse symptoms to marijuana exposure in the years following legalization. Such data are only available for Colorado, where the number of ED visits for marijuana exposure increased from a rate of 22 per 100,000 people in 2012 to 38 per 100,000 in the first half of 2014 when retail marijuana sales first began in the state. Out-of-state visits to the emergency room (ER) for marijuana-related symptoms accounted for 78 of every 10,000 ER visits in 2012, compared to 163 for every 10,000 visits in 2014, indicating an increase of 109%. Among Colorado residents, the number of marijuana-related visits was 70 for every 10,000 in 2012 compared to 101 for every 10,000 in 2014, signifying a 44% increase.

While this might have initially given rise to concern, factors such as a person’s reluctance to seek help when criminal penalties existed for marijuana possession might help explain why these numbers were lower pre-legalization. An ongoing assessment of marijuana-related calls to poison centers and visits to EDs is necessary as new consumers become more familiar with marijuana’s effects and legal issues. 

The legalization of marijuana does not help the situation as drug-related ER visits are still on the rise even after the legalization of marijuana.

Based on the drug-related ED statistics (Colorado), marijuana proved the least in terms of patient numbers. This indicates that alcohol was the highest in terms of the patients admitted to the emergency facilities as compared to marijuana in Colorado. 

Moreover, states that legalized marijuana enjoyed a short period of reduced cases of opioid overdose and related ED visits. According to Bachhuber M et al., deaths from opioid overdoes and other substances reduced between 1999 and 2010 [15]. This reduction is associated with marijuana decriminalization. Washington and California had already enacted marijuana laws before 1999. After 1999, states such as Colorado, Maine, Alaska, Nevada, and Massachusetts among others, enacted cannabis use laws. These states recorded a 24.8% decrease in opioid overdose mortality, and these states got stronger after every year of legalization [15].

Shi argues that there has been a sharp increase in marijuana use in states where the drug is decriminalized. After the legalization of weed, hospitalizations from opioid pain reliever (OPR) have also increased. Shi found a 300% increase in hospitalizations associated with cannabis and opioids. However, Shi also reports that OPR abuse and hospitalizations had also reduced by 23% after the legalization of marijuana [16]. The 300% increase accounted for a combined hospitalization of marijuana and OPR overdose or abuse. Otherwise, independently, OPR abuse had reduced because of the legalization of cannabis. Livingston et al., also argued that drug overdose-related deaths had reduced by 0.7 every month after the legalization of cannabis [17]. Based on these statistics, one could argue that states that had legalized marijuana have enjoyed fewer and fewer cases of drug-related ER visits. However, various scholars, who argue that legalizing marijuana contributed to increased drug-related ER visits, challenge the credibility of these statistics.

Vivolo-Kantor et al. argued that cases of drug-related hospitalization have increased in states such as Maine and Nevada [18]. According to a study done in 2016-2017, these states had a 34.5% increase in drug overdose hospitalization cases. Further studies by Weiss et al. found that states such as Washington, D.C., Massachusetts, California, Nevada, and Alaska had recorded increased ER department visits associated with a drug overdose [19]. These studies were comparing ER visits for the period between 2009 and 2014. These studies found that the entire United States had witnessed a 64.1% increase in drug-related (especially OPR) ER visits. According to Weiss et al., Washington recorded a 60.1% increase, Massachusetts witnessed a 71.1% increase, and California had a 79.0% increase, while there were no stats for Nevada and Alaska [19]. In 2009, there was no comparison. However, in Nevada, out of 100,000 visits to the ER in 2014, 183.1 were associated with a drug overdose.

These states recorded the highest number of drug overdose cases in the country. This is despite the arguments that cannabis legalization was contributing to decreased cases of drug and substance dependency and overdose. These studies are contradictory as one argues that the legalization of cannabis reduces cases of opioid use. On the other hand, statistics show that cases of a drug overdose in the states that decriminalized marijuana are on the rise. Therefore, there is a need for further research on the relationship between the legalization of weed and drug and substance overdose. However, this study argues that drug use and overdoses are on the rise and does not find substantial evidence of an association between marijuana legalization and drug-related ER visits.

Part 7: Alcohol- and drug-induced death and suicide rates**

According to reports by the Centers for Disease Control and Prevention (CDC), about 115 individuals lose their lives each day in the US due to causes that are related to opioids [20]. In a study by Shovera, Davis, Gordon, & Humphreys, researchers concluded that there was an increase in the availability of cannabis in states where its use for medical purposes had been legalized [21]. They also indicated that individuals in these states resorted to substituting cannabis for opioids for either intoxication purposes or pain management. They also concluded that such substitutions were occurring at levels sufficient to cause a significant overdose that would be fatal within the population. Their results indicated that states that had laws that were comprehensive on medical cannabis use still had significant levels of mortalities due to opioid overdose. These back the data depicted by the CDC Wonder online database that the legalization of cannabis in the US has increased drug-related deaths.

According to the data obtained from the CDC Wonder online database on the underlying causes of death in the states of Alaska, California, Colorado, Maine, Massachusetts, Nevada, Oregon, and Washington and Washington, D.C., the rate of deaths associated with drugs has significantly increased [22]. The general implication of this data is that the number of deaths recorded as a result of substance use has increased. The legalization has been on two fronts. In 2001, the state of Colorado legalized marijuana for medical use, and this was extended to recreational use in 2012. Washington and Oregon, on the other hand, legalized it for medical use in 1998 and recreational use in 2012 and 2014, respectively. According to the reports by Hansen, Miller, & Weber, the use of marijuana for recreational purposes has increased deaths due to traffic accidents by 92% in Colorado and 28% in Washington, a further testament to the increase in deaths due to this drug [23].

The drug-related death rate in Alaska in the year 2007, before the legalization of marijuana, was 12.1 per 100,000. In the year 2018, following the legalization, the rate was 16.5. This implies an increase in drug-related deaths following legalization. In California, the rate increased from 9.0 to 11.9, while in Colorado, the death rate due to drug-related issues increased by 4.7. In Maine, during the same period, there was an increase in drug-related rate by 10.4, while Massachusetts had an increase of 14 from the previous value of 11.5. This indicates an increase in more than twice the initial rate of drug-related deaths recorded in 2007. In Nevada, the rate increased by 4.3, Oregon increased by 2.5, and Washington increased by 2.2, while Washington, D.C. had an increase in the rate of deaths by 11.2. Table 7 summarizes this data. 

**Table 7:** Drug-related death rates per state Rate per 100k Source: [22] CDC, Centers for Disease Control and Prevention

State of interest	Legalization Date	Rates in 2007	Rates in 2018
Alaska	2015	12.1	16.5
California	2016	9.0	11.9
Colorado	2012	11.5	16.2
Maine	2017	11.2	21.6
Massachusetts	2016	11.5	25.2
Nevada	2017	16.7	21.0
Oregon	2015	10.1	12.6
Washington	2012	12.4	14.6
Washington, D.C.	2015	16.0	27.2
US value	n/a	9,4	16,9

According to the United States of America Drug Abuse Warning Network, cannabis use contributes to a significant number of visits to EDs of hospitals due to suicide attempts [24]. It is estimated that 6.5% of all suicide attempts that are related to drugs are due to cannabis. In all suicide cases, toxicology reports indicated that 16.8% of patients tested positive for cannabis. A literature review conducted by Borges, Bagge, & Orozco indicated that about 9.5% of reports by the toxicologist had cannabis as the main substance in the patient’s system at the time of suicide [25].

In another cross-over study carried out in Mississippi involving 363 participants who had made recent attempts at suicide and had received treatment at a hospital, researchers made a comparison of rates of the use of cannabis within 24 hours prior to the suicidal attempt with the use of cannabis within 24 hours of the previous day to the day they attempted suicide. Their findings indicated that 10.2% of all participants had consumed cannabis during the case period, which was within 24 hours before the suicidal attempt. Approximately 13.2% had taken cannabis during the 24 hours before the day of the attempt [24]. Our findings are summarized in Table 8.

**Table 8:** Suicide rates in states with legal marijuana Age-adjusted number of deaths due to intentional self-harm per 100,000 population Source: [22]

State of interest	Suicide Rates (2012)	Suicide Rates (2018)
Alaska	23,3	25,5
California	10,8	11,1
Colorado	17,2	21,0
Maine	13,4	16,3
Massachusetts	8,9	9,0
Nevada	20,5	22,2
Oregon	17,5	18,5
Washington	14,1	15,5
Washington, D.C.	6,8	5,5
US Rate	12,4	13,9

Data indicates a general increase in the rate of suicides in Alaska, California, Colorado, Maine, Massachusetts, Nevada, Oregon, and Washington, except Washington, D.C. which recorded a decrease in suicides following the legalization of marijuana [22]. According to the World Health Organization (WHO), the use of cannabis is associated with schizophrenia and psychosis [24]. Schizophrenia is a mental health condition characterized by a distorted sense of an individual’s self and his perception, emotions, behavior, and thoughts. The consumption of cannabis that has a high concentration of THC results in psychotomimetic and anxiogenic effects. These effects increase the risk of suffering from psychotic disorders including schizophrenia. In fact, the use of cannabis with high levels of THC and low concentration of cannabidiol (CBD) increases the likelihood by three to five times of suffering from schizophrenia [26]. Additionally, studies have indicated that cannabis use increases negative symptoms in individuals suffering from psychosis [24]. Both psychosis and schizophrenia are associated with an increase in suicidal tendencies [27]. Other than the above, depression is associated with cannabis use. Depression is characterized by a feeling of worthlessness that increases suicidal tendencies of patients suffering from these mental conditions.

Part 8: Marijuana revenues and other financial aspects

Reduced marijuana arrests have saved the judiciary and law enforcement substantial amounts of money. For example, California used approximately $200 million to enforce marijuana laws from 2000 to 2010. Several states allocate marijuana tax revenue to reimburse regulatory agencies who oversee the regulations laid out by state laws. Prioritizing the reimbursement of regulatory agencies ensures that the marijuana industry’s revenue pays the administrative costs of state governments. The remainder of revenue earned from the marijuana industry is allocated differently in various states. Education and public health institutions benefit largely from marijuana taxes. Colorado has generated nearly $600 million from marijuana sales since 2014. The Colorado Education Department got an allocation of $230 million between 2017 and 2015 to help in funding the construction of schools, early literacy, prevention of bullying, and health programs that emphasize behavior. Oregon allocates 40% of its revenue earned from marijuana sales to the funding of schools in the state and has provided $34 million to school funding so far. Nevada allocates a 15% wholesale tax of its revenue to fund schools [1].

Moreover, drug treatment and state alcohol funds benefit largely from marijuana taxes. For instance, Oregon distributes 20% to the treatment of drug and alcohol, while Washington allocates 25% to the treatment of substance use disorders, prevention, and education. Additionally, Washington allocates 55% of its revenue from marijuana taxes to the funding of basic health programs. The Alaska Revenue Department has estimated to collect approximately $12 million yearly, which will be used in funding drug treatments and centers of the community residence. Other states, which will begin the licensing of marijuana in 2018, are dedicated toward supporting the treatment of substance use disorders. For example, California will allocate 60% of its revenue earned from marijuana taxes to the funding of prevention of drug use by youth and the treatment of substance use disorders. Moreover, 20% of the tax revenue is allocated to support environmental restoration. 

In Massachusetts and California, revenues generated from marijuana sales are imperatively invested in communities that are most negatively affected by incarceration and drug arrests. Massachusetts and California allocate these funds to support jail diversion, vocational training, economic development, job placement and occupational retraining programs, treatment of mental health, and restorative justice and legal services which make it easier for marijuana convicts to reenter the community after incarceration. Table 9 provides additional information about marijuana revenue allocations.

**Table 9:** Marijuana revenue allocation by state Retail marijuana sales are not permitted in D.C. and there are no plans to allow them in the near future. Thus, D.C. is excluded from the table. Source: [1]

State	Allocation
California	Administrative costs reimbursed, Off-the-top disbursements to research, California Highway Patrol and community reinvestment 60%: youth treatment fund 20%: local government 20%: environmental restoration
Colorado	15% excise tax on wholesale retail marijuana; $40 million to school construction; Remainder to Public School Fund; 15% sales tax on retail marijuana: 10%: Local government 90%: State government (beginning 2018-2019) will be split three ways: (1) $30 million off-the-top to the Public School Fund; (2) 28.15% to the General Fund; and (3) 71.85% to the Marijuana Tax Cash Fund Regular 2.9% state sales tax on medical marijuana Marijuana Tax Cash Fund, which funds health care, monitoring health effects of marijuana, etc.
Washington	Administrative costs reimbursed 25%: Substance use treatment, education, and prevention; 1%: Marijuana-related research at the University of Washington and Washington State University; 50%: State basic health plan trust account; 5%: Community health centers for primary health and dental care services; Remainder: General fund
Oregon	Administrative costs reimbursed 40%: State School Fund 20%: Mental health, alcohol, and drug treatment 15%: State police 10%: Cities, based on population and number of licenses 10%: Counties, based on canopy size+#
Alaska	50%: Programs aimed at reducing recidivism 50%: General fund
Nevada	Administrative costs reimbursed; wholesale tax revenue goes to schools; excise tax revenue goes to rainy day fund
Massachusetts	Administrative costs reimbursed; remaining funds expended for (1) public and behavioral health, including substance use prevention and treatment; (2) public safety; (3) municipal police training; (4) Prevention and Wellness Trust Fund; (5) programming for restorative justice, jail diversion, workforce development, and mentoring services
Maine	Legislation has not yet been introduced to implement sales of adult use of marijuana and define marijuana tax collection and revenue allocation.

Sales in most states that have legalized the regulated use of marijuana have slowly kicked off as the consumers and regulators are adjusting to the new systems. In most states, the general sales and tax revenues have exceeded the projected amounts. Medical marijuana consumption and regulation have raised several questions among people across the country. Sales of medical marijuana have remained constant despite predictions that retail marijuana legalization would negatively affect the sales of medical marijuana.

In Washington, the projected sales were $162 million annually in the first two years. In the first fiscal year, it collected $65 million failing to reach the estimates but after that exceeded the estimates with $185 million and $315 million in the second and third fiscal years, respectively. In Colorado, marijuana has generated about $600 million since 2014 as revenues doubled in the second year and almost tripled in the third. Oregon has also surpassed the estimated sales although it fell short in the first year by around $10 million. From this analysis, it is clear that the regulated sale and consumption of marijuana has economically benefited the states, which have legalized it from the tax revenues they collect.

A balance between producing adequate income to offset state and local governments for controlling marijuana and preventing heavy marijuana consumption is required to establish the optimal tax rate for marijuana. Heavy taxing could lead to consumers being stimulated to purchase marijuana from the unregulated market where marijuana is not taxed [28]. 

Washington, Colorado, Alaska, and Oregon have had to amend their tax rates after marijuana sales began [1]. Washington had implemented a complicated tax structure on marijuana at first but switched to a better one, a 37% excise tax [1]. In Colorado, lawmakers approved a reduction of tax rates in 2016 but reversed it in 2017, increasing tax rates on exclusive marijuana sales. Oregon also switched from weight-based wholesale tax to retail tax. Nevada has learned from these states and implemented an excise tax on retail sales. Across all these states, medical marijuana remains less taxed than retail marijuana, and in Oregon, it is not even taxed at all. The tax structure should focus more on retail sales as a wholesale tax may encourage the rise of unregulated markets. However, medical marijuana should be duty-free across all the states. 

It is estimated that the legal marijuana industry has employed 165,000-230,000 workers across the country, either part or full time. This number continues to grow with the continued legalization of marijuana in other states to replace the existing unregulated markets. Growth in employment is a result of the significant reduction of unregulated markets and not necessarily from increased demand. Table 10 provides additional information on the financial aspects of marijuana legalization.

**Table 10:** Financial aspects of marijuana legalization in various states Source: [1], [29-30]

State	Employment rates
Colorado	The legalization of marijuana in Colorado created 18,005 full-time jobs in 2015 alone. Most of the jobs (12,500) were directly related to the marijuana business like stores, cultivation, dispensaries, and manufacturing opportunities. Other ancillary jobs, more than 5,000 include security, legal, and consulting services.
Washington	It was estimated that the business had employed 10,894 workers by the end of 2016. More workers were employed on a full-time basis, and their wages had increased by 63% in 2016.
Oregon	The state reports that the industry had created 12,500 jobs associated with cannabis distribution.

According to these findings, it is clear that states that have legalized regulated marijuana markets have created thousands of jobs for the locals. Therefore, the legalization of marijuana has helped curb unemployment in these states sustaining their economy.

Other aspects of marijuana use

Using cannabis in public was not allowed in any of the states that have legalized marijuana for adults aged ≥21 years. It is a misdemeanor in Nevada and Washington, D.C., while in the rest of the states where adult use of marijuana is legal, public use is subject to fines and fees. This issue creates a fundamental challenge to fair marijuana enforcement because they disproportionately burden the poor with financial sanctions [31].

These concerns may be addressed by businesses being permitted to legally allow marijuana consumption in their premises as it would enable people to consume marijuana in safe places.

Interestingly, African Americans encounter disproportionate enforcement of public marijuana consumption laws. Communities affected through marijuana criminalization continue struggling to overcome insurmountable barriers, which limit their participation in the legal market. Due to this, various cities and states have implemented policies and rules to reduce the barriers limiting entry to the marijuana industry, increase equity, and remedy the earlier harm earlier to the marijuana market and industry. The equity programs aim at increasing the representation of the people who are affected most by marijuana criminalization such as Latin Americans and African Americans. For instance, Oregon allocates some of its marijuana tax revenue to help finance women-owned and minority-owned businesses that sell marijuana [32]. Having a past marijuana conviction may affect the chances of obtaining licenses to potential business investors. To avert this problem, some states such as Colorado, California, and Oregon have allowed people previously convicted of marijuana offenses to change their criminal records retroactively. 

Real equity is impossible unless low barriers to enter the marijuana business are introduced, and start-up capital is provided to low-income persons through the availability of small business grants or loans. California reduced many marijuana penalties, as all penalties for underage youth were reduced and instead treated as infractions. Moreover, all individuals with marijuana business licenses have to adhere to environmental regulations. While marijuana legalization led to reduced arrests of the latinx and blacks for marijuana offenses, racial disparities continue to persist. For instance, in Alaska, the whites and blacks experienced a decline in arrests for marijuana offenses, but disparities still remain. In 2016, 29% of the 17 arrested persons in Alaska were blacks. Considering blacks constitute only 4% of Alaska’s population implies that their arrests are ten-fold that of whites [1]. 

A similar pattern has emerged in Washington, D.C. Marijuana arrests in D.C. illustrate a tendency of increase in the arrests for public consumption; the vast majority targets black men.

This is despite the fact that black residents only make up around 49% of D.C.’s population, with the rate of marijuana use being similar to that of white residents [33]. Police departments should adopt policies that prohibit police officers from practicing racial profiling while educating them on the harms of racial discrimination. Moreover, police departments should be accountable and transparent. 

States where marijuana use is Illegal

In this section, we have examined the possible impact of illegalization of marijuana on its use and subsequently, the society. We have analyzed the trends in the past few years and compared them with the current rates. 

Several states have discriminated against marijuana and have made its use illegal. The possession, sales, and use of a small amount of pot can lead to imprisonment or fines because marijuana is entirely illegal in some states in the US. As mentioned earlier, some states have legalized the use of marijuana, but the federal government has maintained its stand that marijuana is illegal by classifying it under Schedule 1 substances having a high potential for abuse [34]. This implies that marijuana is considered to have no medical significance and a high likelihood of abuse. The classification puts marijuana in the same class as heroin and a more restrictive category when compared to Schedule 2 drugs such as methamphetamine and cocaine. Despite federal prohibition, the federal government has taken a relaxed approach toward marijuana, generally allowing the states to decide about marijuana legalization [34]. This has made several states legalize marijuana for recreational and medical purposes, while others have maintained their position that marijuana should not be legalized. 

Analysis of the states where marijuana use, possession, and/or sale is illegal was done based on the following criteria:

· The use of marijuana among the youth in the previous years compared to the current situation

· The rate of violent crimes in the previous years compared to the current rate

· The rate of fatal accidents and car crashes in the previous years compared to the current rate

· The rate of admission for substance abuse in the treatment facilities 

The rate of use of marijuana in the illegalized states is expected to differ among the states. This may result from the implementation of strict laws and regulations surrounding the use, possession, and sales of marijuana. It may also be based on the fact that some states allow the use of marijuana for medical purposes only while others completely illegalize marijuana. 

Almost two-thirds of the US have legalized marijuana. However, it is important to note that in 14 out of the remaining 18 states where broad-based medical pot is to be legalized, laws have been passed to allow the use of CBD, particularly in special cases or selected diseases or ailments. CBD is treated as a non-psychoactive cannabinoid known for its alleged medical benefits. Tennessee is one of the 18 states where the use, possession, or sale of marijuana is completely illegal, whether for medical or recreational purposes. However, it allows for the sale of CBD oil extracted from the hemp plant. Hemp is normally known for its richness in CBD content with a little amount of THC that is known to make a user get 'high'.

Some of the states that have not legalized the use of marijuana include Idaho, Wyoming, South Dakota, Nebraska, Kansas, Iowa, Wisconsin, Texas, Indiana, Kentucky, Tennessee, Mississippi, Alabama, Georgia, South Carolina, North Carolina, and Virginia, out of which four states, including Kansa, Nebraska, Idaho, and South Dakota, have completely banned all forms of cannabis and its cannabinoids, including CBD [35].

State of marijuana use in Texas

Texas is one of the states that have not enacted laws to legalize marijuana use entirely. Marijuana is considered a significant drug threat to Texas. The drug is available and widely abused throughout Texas despite its discrimination. According to Born-Miller, around 74% out of the 165 law enforcement respondents in Texas who rated their level of marijuana abuse in their departments reported a high level of abuse of the drug [36]. Approximately 22% of the law enforcement departments reported a medium level of marijuana abuse. This brings to question the significance of the illegalization of marijuana in the states. The rate of abuse of marijuana in Texas is relatively high even though the state prohibits marijuana use [37]. Regardless of the high rate of marijuana abuse within the state, data indicate the number of Texas residents aged ≥12 years who have reportedly used marijuana at least once in a month [37].

The rate of abuse of marijuana in Texas is a significant concern for the health providers in the state. According to the Texas Commission on Alcohol and Drug Abuse (TCADA), the percentage of adult hospital admissions resulting from marijuana abuse is fluctuating, but from 2002 to 2006, there was an increase in the overall percentage. The number of admissions in 2002, 2003, 2004, and 2006 was 3,083, 3,729, 3,432, and 4,296, respectively [38]. The abuse of marijuana by adolescents is of great concern as well, particularly to health and law enforcement professionals. TCADA reports that marijuana was the primary drug of abuse for around 82% of the 5,278 youth admissions for treatment to the health facilities funded by TCADA in 2002 [38].

Additionally, marijuana has been described by 7th to 12th grade students as a frequently abused illicit drug in Texas based on the 2002 Texas School Survey of Substance Abuse Among Students from Grades 7 to 12 [38]. The survey indicated that the average age of first-time use of marijuana in Texas is 13 years [39]. Marijuana is most commonly detected among adult male arrestees in Texas. Around 32.9% of adult males arrested in 2009 in Texas tested positive for marijuana [38].

Marijuana Use Among the Youth in Texas

Since 2002, the percentage of adolescents who report the use of marijuana has decreased. It is also reported that more young adolescents report relatively stronger disapproval of marijuana use. This is according to the new research conducted by the University of Texas at the Austin School of Social Work. The results offer guidance to educators and policymakers who look into marijuana use. Fundamental changes were marked among young adolescents between the ages of 12 to 14 years; a 15% decline, that is, from 6% in 2002 to 4.5% in 2013, in the number of young adolescents who reported using marijuana in the past 12 months was registered [40]. At the same time, an increase in those reporting strong disapproval of marijuana use initiation from 74% to 79% has been documented.

The results may suggest that the recent changes in public policy such as the discrimination, legalization, and medicalization of marijuana in states and cities across the country have not led to more use or higher approval of marijuana use among the youth. Among adolescents, no significant difference was observed concerning the trends in the disapproval of marijuana. However, the percentage of older adolescents who reported the use of marijuana during the previous year reduced from 26% in 2002 to 22% in 2013 [41]. A different pattern was, however, noted among young adults between the ages of 18 and 25 years. The study showed a downward trend in marijuana use disapproval among young adults with about 41% reporting strong disapproval in 2002 and 23% in 2013. The recent changes in policy and the increased exposure to marijuana use may have an influence on the emotional state of the youth regarding those using it. However, this does not necessarily impact their use. From this result, it can be deduced that besides the high rate of marijuana use among American youth, fundamental changes are underway regarding the perception and use of marijuana among them.

Marijuana-related Arrests in Texas

Even though the attitude across the country seems to change with certain states even decriminalizing marijuana use, the arrests for possession of marijuana are still common in Texas. A recent study done by the American Civil Liberties Union indicates that Texas has one of the highest rates of arrests for the possession of marijuana in the country [42]. In 2010, Texas was recorded as the second-highest in the country concerning the number of those being arrested for possessing marijuana. Around 74,000 people were arrested for possession of marijuana in 2010. In addition to the high arrest rates in Texas, the study also indicated a racial disparity among those charged with marijuana possession. Marijuana arrests today account for more than half of all drug arrests in Texas. Among 200,087 arrests between 2001 and 2010, around 88% were simply for the possession and use of marijuana. The arrest statistics indicate a crucial trend, that is, significant racial bias. Even though there are roughly equal usage rates, blacks had 3.73 times higher likelihood of being arrested for marijuana possession or use compared to whites. Although black Americans make up only around 12% of the entire Texas population, the report indicated that they accounted for more than 25% of those who faced marijuana possession charges in 2010 [43]. These data are summarized in Table 11. 

**Table 11:** Texas marijuana arrests Source: Federal Bureau of Investigation, Uniform Crime Reporting Program

Texas	2014	2015	2016
Possession	66,060	59,758	63,599
Sales	1,524	1,475	1,350
Total	67,584	61,233	64,949

Marijuana-related Fatal Accidents in Texas

As mentioned earlier, marijuana possession remains illegal under the Texas Federal Law. It is also against the law to possess marijuana for recreational use. However, some still manage to sneak in and sell marijuana, contrary to the law. A survey conducted in Texas indicates that around 70% of cannabis consumers have reported driving while impaired at least once in the previous year. In 2018, police reported an increase in car collisions or crashes associated with marijuana use. The crash rates were reported to have increased by an average of 5.2% in Texas [44]. 

Marijuana-related Hospitalizations in Texas

Across Texas, the study indicates that between 2004 and 2011, emergency department visits involving the use of marijuana had increased from 51 to 73 visits for every 1,000 patients. The percentage of hospital admissions of marijuana combined with other drugs also increased from 63 to 100 visits during this period for every 1,000 patients [45]. Researchers depended on hospital records which could not identify marijuana use as the cause of the visit but just a contributing factor. In Texas, marijuana has caused an increase in ER visits, particularly by those who have used marijuana. Many people who have habitually or recently used cannabis are visiting the hospital, even if that was not the primary condition. The major risks resulting from the use of marijuana often result from the risky behaviors that people engage in after using it such as driving, rather than any associated toxic effects of the use of the substance. Marijuana-related ER visits have thus increased in several hospitals across Texas, and over the last decade, hospitalization due to marijuana use has increased in Texas and across the nation. 

According to the statistics indicated in Table 12, the percentage of marijuana users has increased between 2002 and 2014. In 2002, 8.5% of marijuana users were recorded, which increased to 9.5% in 2014. The percentage of marijuana users, although fluctuating in this period, has shown an increasing trend.

**Table 12:** Percentage of past-year marijuana use* among all persons aged >12 years, by age group – National Survey on Drug Use and Health, various states, 2002-2014 Source: National Survey on Drug Use and Health, 2002–2014. CI, confidence interval *Past-year marijuana use is defined as those who reported the use of marijuana within 12 months preceding the date of interview; these also include those who reported the use of marijuana within 30 days preceding the date of interview.

Texas, 2002-2014												
Age group (years)	2002-2003	2003-2004	2004-2005	2005-2006	2006-2007	2007-2008	2008-2009	2009-2010	2010-2011	2011-2012	2012-2013	2013-2014
	% (95% CI)	% (95% CI)	% (95% CI)	% (95% CI)	% (95% CI)	% (95% CI)	% (95% CI)	% (95% CI)	% (95% CI)	% (95% CI)	% (95% CI)	% (95% CI)
Total	8.5 (7.7-9.5)	8.6 (7.9-9.4)	8.6 (7.8-9.5)	8.4 (7.5-9.3)	7.9 (7.1-8.9)	7.9 (7.0-8.8)	8.3 (7.5-9.3)	9.2 (8.3-10.2)	9.3 (8.4-10.4)	9.4 (8.5-10.4)	9.4 (8.5-10.3)	9.5 (8.7-10.3)
12-17	13.8 (12.3-15.5)	13.4 (11.9-14.9)	12.0 (10.6-13.5)	11.3 (9.9-12.9)	11.3 (9.8-12.9)	11.0 (9.6-12.7)	11.0 (9.7-12.5)	12.2 (10.7-13.9)	12.2 (10.8-13.8)	12.4 (10.8-14.1)	11.1 (9.8-12.5)	10.7 (9.3-12.2)
18-25	22.0 (19.9-24.4)	21.8 (19.5-24.2)	22.5 (20.2-24.9)	22.0 (20.0-24.2)	20.1 (18.1-22.2)	20.3 (18.3-22.4)	22.5 (20.4-24.6)	24.0 (21.9-26.1)	24.1 (21.8-26.6)	24.7 (22.4-27.2)	25.3 (23.5-27.3)	25.6 (23.3-27.9)
≥26	5.1(4.2-6.1)	5.3 (4.5-6.2)	5.3 (4.5-6.4)	5.2 (4.3-6.3)	5.1 (4.2-6.2)	5.1 (4.1-6.2)	5.3 (4.4-6.3)	6.0 (5.0-7.2)	6.1 (5.0-7.4)	6.1 (5.0-7.3)	6.1 (5.2-7.2)	6.3 (5.5-7.3)
Nebraska, 2002-2014												
Age group (years)	2002-2003	2003-2004	2004-2005	2005-2006	2006-2007	2007-2008	2008-2009	2009-2010	2010-2011	2011-2012	2012-2013	2013-2014
	% (95% CI)	% (95% CI)	% (95% CI)	% (95% CI)	% (95% CI)	% (95% CI)	% (95% CI)	% (95% CI)	% (95% CI)	% (95% CI)	% (95% CI)	% (95% CI)
Total	9.6 (7.8-11.8)	9.0 (7.2-11.4)	8.6 (6.8-10.9)	9.0 (7.6-10.5)	8.0 (6.6-9.7)	8.3 (6.6-10.4)	9.3 (7.2-11.8)	9.4 (7.8-11.3)	9.1 (7.5-11.1)	9.3 (7.6-11.3)	9.7 (7.8-12.0)	9.8 (8.0-11.8)
12-17	17.5 (13.7-22.0)	13.9 (10.8-17.7)	10.7 (10.8-17.7)	9.7 (7.2-13.0)	9.2 (6.5-12.7)	11.5 (8.4-15.4)	10.5 (7.8-14.0)	10.1 (7.5-13.6)	11.0 (8.7-13.7)	13.1 (9.8-17.2)	14.7 (11.9-18.2)	11.9 (8.8-15.9)
18-25	25.6 (20.6-31.2)	23.0 (18.1-28.7)	22.3 (18.0-27.4)	25.1 (21.2-29.6)	25.4 (21.2-29.6)	23.6 (19.1-28.8)	24.2 (19.9-29.2)	25.9 (22.2-30.0)	24.9 (21.3-28.9)	26.0 (22.7-29.7)	28.5 (24.4-32.9)	29.7 (24.5-35.3)
≥26	5.4 (3.7-8.0)	5.6 (3.7-8.4)	5.6 (3.7-8.5)	5.7 (4.3-7.6)	4.5 (3.1-6.3)	5.0 (3.4-7.3)	6.3 (4.2-9.3)	6.1 (4.2-8.7)	5.9 (4.2-8.3)	5.8 (4.0-8.3)	5.7 (4.0-8.2)	6.0 (4.4-8.1)
Idaho, 2002-2014												
Age group (years)	2002-2003	2003-2004	2004-2005	2005-2006	2006-2007	2007-2008	2008-2009	2009-2010	2010-2011	2011-2012	2012-2013	2013-2014
	% (95% CI)	% (95% CI)	% (95% CI)	% (95% CI)	% (95% CI)	% (95% CI)	% (95% CI)	% (95% CI)	% (95% CI)	% (95% CI)	% (95% CI)	% (95% CI)
Total	9.1 (7.6-10.9)	8.8 (7.1-10.9)	8.9 (7.2-10.9)	8.7 (7.1-10.7)	8.0 (6.5-9.7)	8.7 (6.9-11.0)	10.5 (8.5-13.1)	13.3 (10.9-16.3)	13.0 (10.2-16.5)	9.4 (7.3-11.9)	9.4 (7.6-11.4)	11.4 (9.4-13.8)
12-17	14.6 (11.5-18.4)	14.1 (10.4-18.7)	12.1 (8.6-16.8)	11.5 (8.6-15.1)	10.8 (8.2-14.1)	12.7 (10.2-15.7)	13.5 (10.9-16.6)	13.8 (10.4-17.9)	14.5 (10.9-18.9)	13.3 (10.4-16.9)	12.6 (9.5-16.6)	15.7 (12.3-19.9)
18-25	23.4 (19.9-27.3)	22.5 (18.5-27.0)	25.2 (20.9-30.1)	25.2 (19.7-31.7)	23.0 (17.0-30.2)	23.4 (17.6-30.5)	27.8 (22.0-34.3)	29.2 (24.5-34.4)	23.1 (18.7-28.2)	20.4 (15.4-26.5)	24.3 (18.8-30.8)	27.4 (22.5-33.0)
≥26	5.2 (3.7-7.4)	5.2 (3.4-7.8)	5.0 (3.4-7.4)	5.1 (3.6-7.2)	4.8 (3.3-6.8)	5.5 (3.6-8.4)	7.1 (4.8-10.3)	10.4 (7.5-14.2)	11.0 (7.7-15.3)	6.8 (4.5-10.1)	6.2 (4.6-8.5)	8.1 (5.9-10.8)
Alabama, 2002-2014												
Age group (years)	2002-2003	2003-2004	2004-2005	2005-2006	2006-2007	2007-2008	2008-2009	2009-2010	2010-2011	2011-2012	2012-2013	2013-2014
	% (95% CI)	% (95% CI)	% (95% CI)	% (95% CI)	% (95% CI)	% (95% CI)	% (95% CI)	% (95% CI)	% (95% CI)	% (95% CI)	% (95% CI)	% (95% CI)
Total	8.3 (6.8-10.2)	8.7 (6.9-10.9)	8.2 (6.6-10.2)	7.5 (6.0-9.3)	7.7 (5.9-9.9)	7.6 (5.9-9.6)	8.3 (6.7-10.3)	8.8 (7.0-10.8)	8.8 (7.0-11.1)	9.4 (7.9-12.4)	9.9 (7.9-12.4)	9.8 (8.2-11.7)
12-17	12.4 (9.5-15.9)	13.2 (10.7-16.0)	12.3 (9.7-15.4)	10.6 (7.9-14.1)	11.6 (8.7-15.4)	10.2 (7.5-13.7)	8.7 (6.7-11.1)	9.9 (7.4-13.2)	11.1 (8.7-14.0)	10.5 (8.0-13.6)	9.4 (7.2-12.3)	8.8 (6.6-11.7)
18-25	24.5 (20.4-29.0)	25.1 (21.3-29.3)	24.3 (20.3-28.8)	20.2 (16.3-24.9)	17.0 (13.5-21.2)	19.6 (15.7-24.1)	22.6 (18.2-27.7)	21.6 (17.8-25.9)	21.8 (18.6-25.3)	22.6 (19.6-25.8)	24.5 (20.2-29.5)	27.8 (22.8-33.3)
≥26	4.9 (3.3-7.2)	5.2 (3.4-7.9)	4.9 (3.3-7.1)	4.8 (3.3-7.0)	5.6 (3.8-8.3)	5.2 (3.6-7.6)	5.8 (4.1-8.3)	6.4 (4.5-9.0)	6.3 (4.3-9.2)	6.9 (4.9-9.8)	7.5 (5.4-10.2)	6.8 (5.2-8.9)

**Figure 8:**
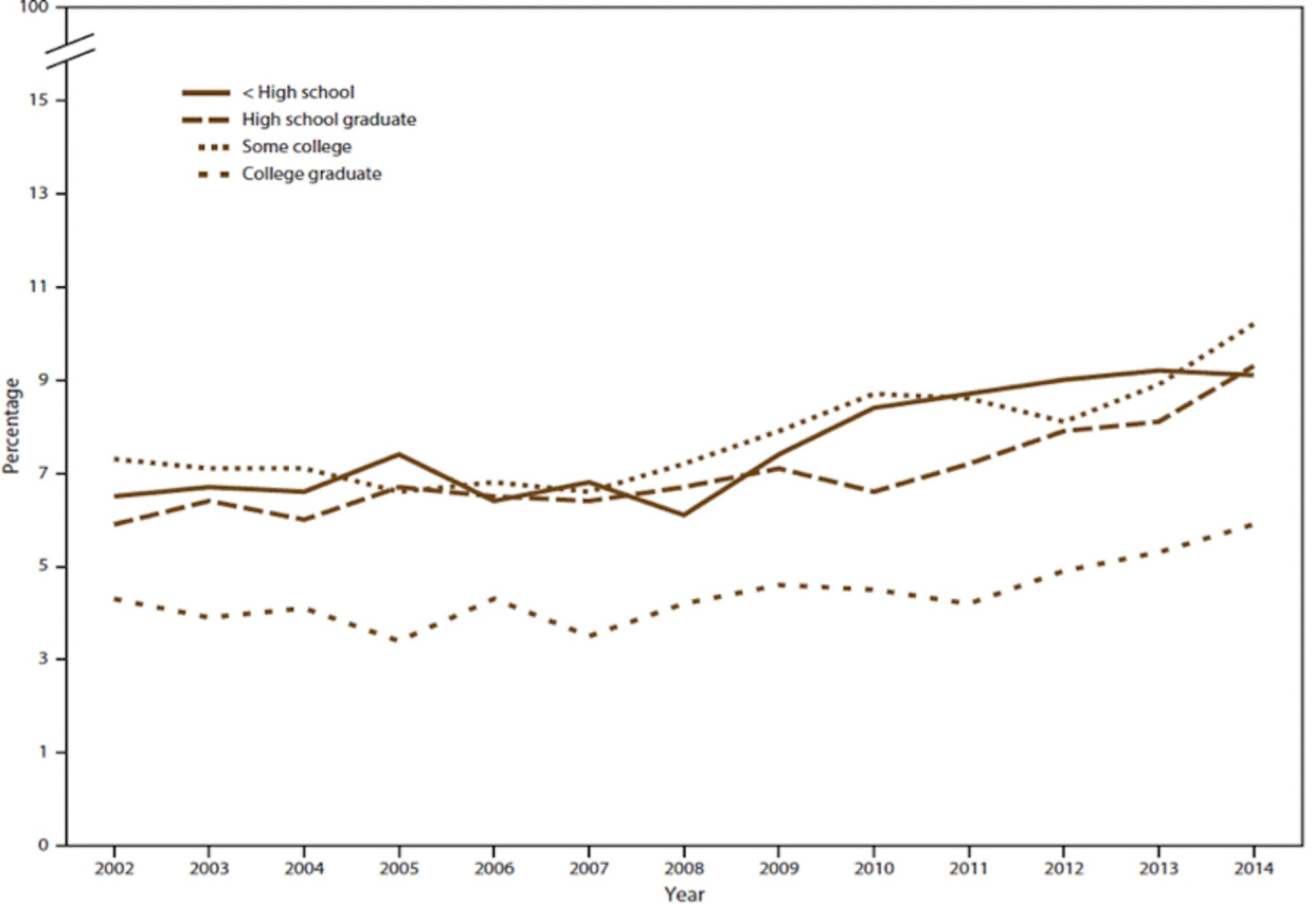
The percentage of marijuana use in Texas among adults from 18 years from 2002 to 2014 Source: Federal Bureau of Investigation, Uniform Crime Reporting Program

In Texas, marijuana, along with alcohol, is the most frequently abused illegal drug. In 2013, around 7.5% of the Texas population aged ≥13 years admitted using marijuana during the previous 30 days. It is reported that from 2002, the use of marijuana in Texas has increased among people aged ≥18 years, but its use among 13 to 17-year-old students has remained relatively the same [38]. This may be attributed to the drop in awareness of the high risk associated with marijuana use, coupled with an increase in the belief of accessibility (it is relatively easy to get marijuana). Few legal consequences for possessing marijuana in the state may also play a crucial part in the increasing rate of use among adults. Figure 8 shows the percentage of marijuana use in Texas among adults of age ≥18 years from 2002 to 2014. It is evident from the figure that there has been an increase in marijuana use in Texas despite it being illegal [37].

State of marijuana use in Nebraska

In the state of Nebraska, marijuana is illegal for all purposes. Possession of marijuana is illegal in this state. Possession of one ounce or less is considered an infraction and a crime punishable by the law with a maximum fine of around $300. Possessing one ounce or less can also be considered a misdemeanor and one can be fined $500 for a second conviction. In case convicted for the third time, one can be imprisoned for a maximum of seven days and fined a maximum of $500. The cultivation of marijuana is not allowed as well [46]. The penalty for cultivating marijuana is based on the total weight of the weed found in the plantation. Surprisingly, Nebraska does not have a plan for medical marijuana or any allowances for patients in the state even though a bill is being presented for legalizing medical marijuana. 

Marijuana Use among the Youth in Nebraska

In Nebraska, the rate of marijuana use among people aged ≥12 years slightly increased between 2002 and 2014. In 2002, 9.6% of marijuana users was recorded in Nebraska. This, however, increased to 9.8% in 2014. Although the percentage of marijuana users fluctuated in this period, 9.8% is the highest recorded percentage. These data indicate that the rate of use of marijuana among youth in Nebraska has widely increased in recent years, which remains a concern because of the health risks associated with marijuana use. The percentages of past-year marijuana use among all persons aged ≥12 years in Nebraska have been summarized in Table 12.

Marijuana-related Hospitalizations in Nebraska

In Nebraska, the rate of marijuana abuse is reported to be very high. Although marijuana is the most commonly abused illicit drug in the state, treatment admissions associated with marijuana abuse to publicly funded healthcare facilities decreased from 1997 to 2001. Nebraska hospitals have experienced an increase in visits by teenagers and young adults testing positive for marijuana. Between 2000 and 2001, the number of marijuana-related admissions to publicly funded healthcare facilities in Nebraska increased from 684 to 862. Another study conducted in Nebraska reported that the rate of marijuana-related visits to the ER by youth increased from 107 in 2005 to 630 in 2014. At the same time, the rate of marijuana visits among total hospitalizations that involved 13 to 21-year-olds increased in Nebraska with around four visits for every 1,000 patients in 2015. According to the NSDUH, 300,000 million adolescents between the ages of 12 and 17 years reported using marijuana [47]. While the federal government continues to treat marijuana as a Schedule 1 narcotic, the use of this drug has increased, particularly in Nebraska. Some of the data mentioned above are summarized in Table 13.

**Table 13:** Drug-related treatment admissions to Publicly Funded Facilities, Nebraska, 1997-2001 Source: Treatment Episode Data Set

Year	Methamphetamine	Cocaine	Marijuana	Heroin
1997	567	537	1,004	40
1998	701	802	901	15
1999	511	512	696	19
2000	902	794	684	12
2001	1,294	757	862	11

Marijuana-related Arrests in Nebraska

Although marijuana use tops the list of drugs abused in Nebraska, research indicates that marijuana abuse in Nebraska is lower than the rates reported across the country. According to a survey done by the Arrestee Drug Abuse Monitoring (ADAM) program, it is indicated that around 48% of the adult males arrested in 2,000 tested positive for marijuana [48]. The number of marijuana-related federal sentences in Nebraska was relatively lower than the number reported nationwide in 2001. Approximately 10% of the drug-related federal sentences in the state was resultant of marijuana abuse compared to the national rate of 33%. It is also indicated that the percentage of federal sentences for the violation of marijuana laws in Nebraska increased from 17 in 1997 to 29 in 2001 [48]. A new study conducted by the University of Nebraska indicates that marijuana arrests in Western Nebraska have increased subsequent to the legalization of marijuana in Colorado for recreational purposes. This study confirms reports from prosecutors and sheriffs that the number of people being arrested and imprisoned for marijuana has increased after a change in the Colorado law. However, researchers do not believe that the change in Colorado law is responsible for the increase in marijuana arrests. It may be argued that increased enforcement efforts by the authorities may result in increased arrests. In general, the rate of marijuana-related arrests in Nebraska increased by 11% between 2013 and 2014, when the recreational use of marijuana was legalized in Colorado.

Additionally, the budget for enforcement efforts for Nebraska was increased by 11% with a total of $10.2 million spent in the enforcement of marijuana laws in the state. During this period, the rates of both marijuana possession and sales increased. However, most of the arrests were for the possession of marijuana. Before the Colorado legalization, possession arrests were stable in Nebraska, but it reached a six-year high in 2014. There is no doubt that Colorado legalization played a role in the increase in marijuana arrests in Nebraska. Some of the data mentioned above are summarized in Table 14. 

**Table 14:** Percentage of drug-related federal sentences by drug type in Nebraska and the United States, 1997-2001 Source: US Sentencing Commission *Represents the percentage of federal sentences that are drug-related Most recent data is unavailable.

	All drugs*	Methamphetamine	Powdered cocaine	Crack Cocaine	Marijuana	Heroin
Nebraska						
1997	50.8	41.4	15.4	17.0	13.8	9.7
1998	49.8	57.3	11.0	28.6	2.9	0.0
1999	71.5	66.6	9.3	14.2	6.5	1.0
2000	65.9	63.2	9.9	15.8	7.1	1.9
2001	59.7	66.3	6.5	16.5	10.4	0.4
United States						
1997	38.7	10.2	25.3	24.4	27.5	9.7
1998	40.2	11.4	23.4	23.9	30.0	8.9
1999	41.0	12.8	22.1	22.9	31.5	8.0
2000	39.8	14.5	22.8	21.4	31.2	7.7
2001	41.2	14.2	22.1	20.4	32.8	7.2

State of marijuana use in Idaho

Idaho is one of the states where marijuana is entirely illegal, and any possession of this drug is subject to criminal charges. Cannabis penalties in Idaho may include up to five years in prison and a fine of up to $10,000. While some states, including the neighboring state of Washington, have recently legalized the use of marijuana for recreational purposes, Idaho is yet to follow suit. Regardless of the current efforts by other states to legalize marijuana use for recreational and medical purposes, the possession and sale of marijuana in Idaho, even for medical purposes, is still prohibited. Idaho possesses some of the harshest marijuana regulations in the country. If found in possession of more than three ounces of marijuana, one could be charged with a felony, and those who have been convicted of selling marijuana may face 10 years imprisonment and a fine of $30,000 based on the level of involvement and previous convictions. The state laws prohibit pot within the state, and any sale, possession, and/or trafficking is illegal. 

Marijuana-related Arrests in Idaho

Despite the strict laws prohibiting the use, sale, or possession of marijuana, statistics by the Idaho Drug Control Update for 2010-2011 indicate that Idaho is one of the top ten states having high rates of marijuana use among people aged ≥12 years [49]. Approximately 9.36% of the Idaho residents reported using marijuana in the past month. The study indicated that marijuana-related admissions increased in 2012. According to the Federal Bureau of Investigation’s Uniform Crime Reporting Program, the rate of marijuana related-arrests in Idaho has increased from 2014 to 2016 [49]. This may be attributed to the increased ease of access to marijuana and the possibility of purchasing it cheaply from various sources in the state. Many residents tend to disregard the effects of marijuana on their health and the state penalties associated with its use, sale, and possession. 

Marijuana-related Hospital Admissions in Idaho

**Figure 9:**
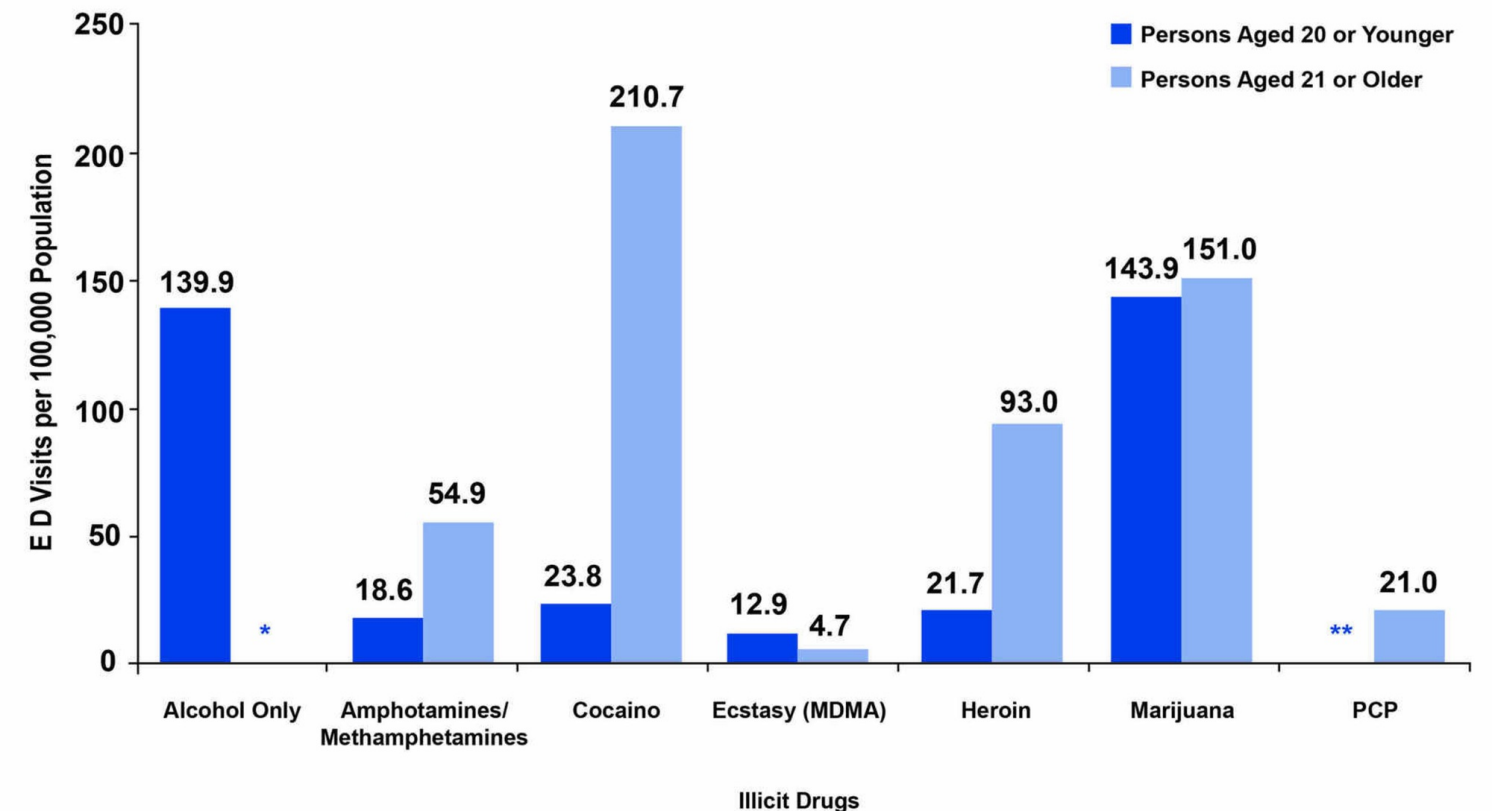
Rates of emergency department visits involving illicit drugs per 100,000 population *Alcohol-only visits are not captured by DAWN for patients of age 21 years or above; **Estimate suppressed due to low statistical precision. Source: 2010 SAMSHA Drug Abuse Warning Network SAMSHA, Substance Abuse and Mental Health Services; DAWN, Drug Abuse Warning Network

In Idaho, marijuana and alcohol are the most abused drugs. Marijuana abuse in the state has been associated with several risks including throat damage, chronic bronchitis, lung damage including cancer, fetal development problems during pregnancy, impairment of attention and motor controls, and impairment of learning capabilities. According to the World Health Organization (WHO), the rate of marijuana-related health complications has increased in the state recently. Figure 9 shows the statistics of drug-related hospital visits in 2010 in Idaho and summarizes our findings. Marijuana scored the second-highest after cocaine for people of age 21 years and above. However, emergency-related hospital visits were the highest for marijuana users of age 20 years and below [49].

Marijuana Use among the Youth in Idaho

Table 12 indicates that Idaho is one of the states wherein the rate of marijuana use has increased significantly despite being illegal. In 2002, marijuana use in Idaho was 9.1%. This rate, however, has increased in 2014 to 11.45%. Between 2002 and 2014, the rate of marijuana use fluctuated with 2010 recording the highest rate (13.3%) followed by 2011 (13.0%). The lowest rate was recorded in 2007 (8.0%).

Fatal Road Accident Involving Marijuana in Idaho

Fatal crashes involving drivers who have recently used marijuana are increasing in Idaho despite the total illegalization of marijuana in the state. Research done in 2012 by the National Drug Survey indicates that the percentage of drivers who are involved in fatal accidents and have recently used marijuana increased from 8% to 17% between 2013 and 2014. One out of six drivers involved in fatal accidents in 2014 had marijuana recently. Marijuana can affect driver safety by impairing vehicle judgment and control. Drivers using marijuana and getting behind the wheel while impaired put themselves and others at risk of accidents. 

State of marijuana use in Alabama

The rate of marijuana use in Alabama increased from 2002 to 2014. In 2002, marijuana users were around 8.3%. This number fluctuated throughout the period up to 2014 when 9.8% of users were recorded. However, it can be noted that 2013 registered the highest number of users at 9.9%. Table 12 summarizes the percentage of past-year marijuana use among all persons aged ≥12 years, categorized by age group, from 2002 to 2014, in Alabama.

**Figure 10:**
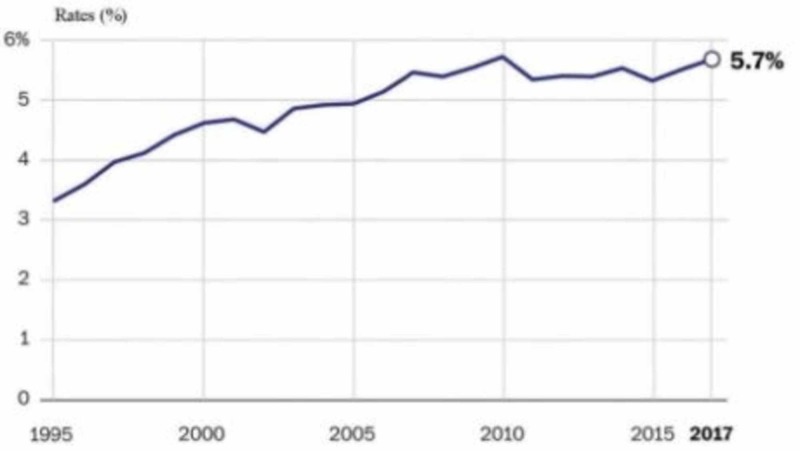
Marijuana possession arrests as share of all arrests reported to the FBI, 1995 to 2017 Source: FBI FBI, Federal Bureau of Investigation

Alabama is one of the states with strict rules for marijuana use. The strict penalties regarding the possession, use, and trafficking of marijuana have been subject to reduction or elimination. In 2014, Carly’s Law was passed to allow federal-approved clinical trials of the use of CBD oil in the treatment of children with a seizure disorder [50]. However, the bill does not entail broad legalization of CBD oil or marijuana use. According to several sources, the number of marijuana-related arrests has increased in Alabama from 1995 to 2017. This has increased the burden of law enforcement officers in the state. Possession of marijuana accounted for approximately 5.7% of all arrests in Alabama in 2017, underscoring the policing level dedicated to controlling the behavior in the state [50]. Figure 10 schematically explains this data.

In 2016, Alabama and its districts spent approximately $22 million to strengthen the prohibition of marijuana possession. Blacks were arrested for misconduct or marijuana possession nearly four times more often, despite studies quoting equal levels of use of marijuana by both black and white men. In 2016, police arrested more people for possessing marijuana (2,351) than for thievery (1,314 arrests) - although that year, 4,557 cases were reported [1]. 

A growing number of states have started treating marijuana like alcohol and tobacco. Meanwhile, Alabama maintains the criminalization of those using marijuana for recreational or medical purposes. Early data demonstrate benefits to public safety and the criminal justice system by this approach. The states that dispensed with arrests for marijuana possession provided police officers an opportunity to focus on the crime of violence. There was also a reduction in the number of arrests for drunk driving. However, evidence of a spike in crime or increased marijuana use among youth is questionable. 

**Figure 11:**
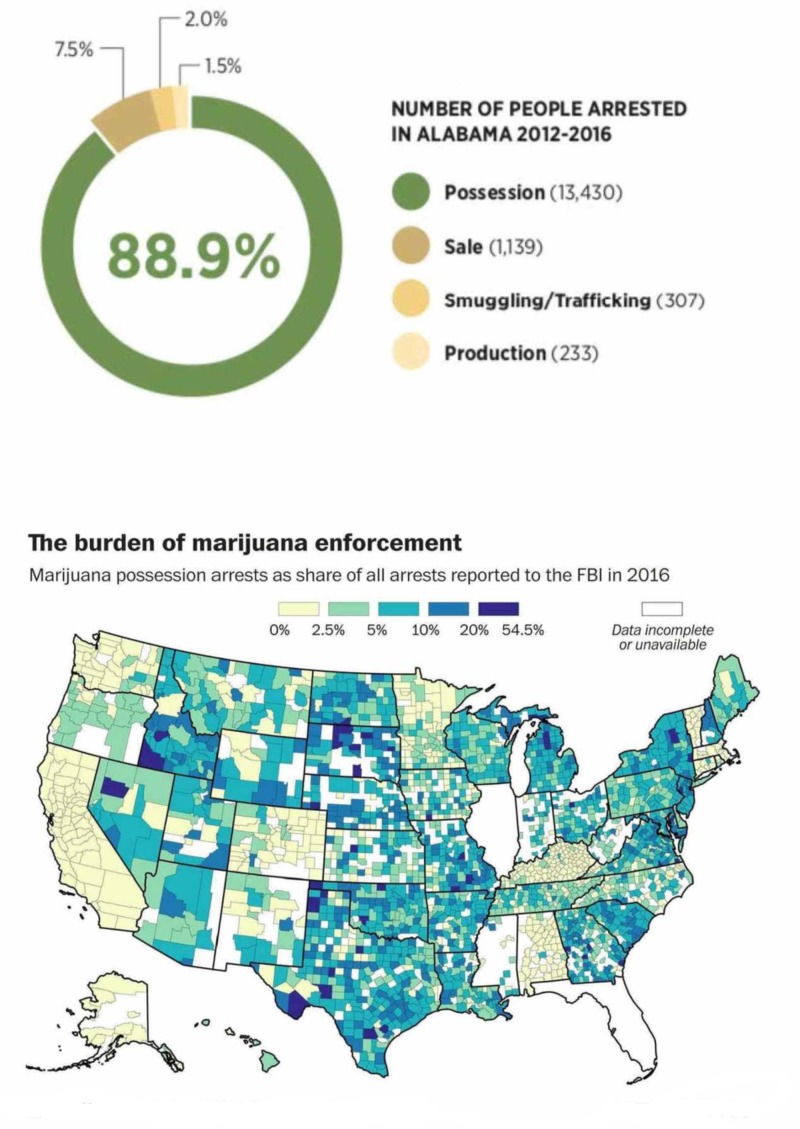
The burden of marijuana enforcement in Alabama vs. USA Source: NACJD analysis of FBI Uniform Crime Reporting Data NACJD, National Archive of Criminal Justice Data

Marijuana laws in Alabama can be interpreted unpredictably. The only marijuana-related crime is drug trafficking with a defined weight threshold (a minimum of 2.2 pounds). Prosecutors can charge a person with possession for personal use, non-personal use, and production or distribution of marijuana totaling less than 2.2 pounds. The same amount of marijuana, possessed by two individuals, can be treated as separate crimes. Figure 11 summarizes these data. 

A total of 585 persons died in Alabama in 2010 as a direct consequence of drug (all substances) use. In Alabama, the rate of drug-induced death (12.2 per 100,000 population) was lower than the national rate (12.9 per 100,000) in 2016. Marijuana has been reported to be the most commonly cited drug among primary drug treatment admissions in Alabama [13]. Alabama State law (Section 32-5A-191) stipulates that “a person shall not drive or be in actual physical contact of any vehicle while under the influence of a controlled substance to a degree which renders him incapable of safely driving,” or while “under the influence of any substance which impairs the mental or physical faculties of such person to a degree which renders him incapable of safely driving.” Although Alabama has imposed a law against drugged driving, law enforcement often lacks adequate tools to enforce and prosecute drugged driving. Unfortunately, a lot of data is still missing. 

Recommendations and suggestions

In the US, the possession and use of marijuana for any purpose is illegal under federal law. Marijuana is classified as a Schedule I substance with the possibility of having a high potential for abuse and no recognized or accepted medical use. In some states, marijuana has been legalized for medical and recreational uses. For instance, marijuana use is legal in the states of Colorado, Alaska, California, Montana, Oregon, Vermont, Hawaii, Nevada, Washington, and Maine. Advocates also believe legalization raises revenue, lowers criminal justice expenditure, improves public health, improves traffic safety, and stimulates the economy. Furthermore, legalization aimed at reducing the crime rate was reported in different states. Critics believe legalization exacerbates marijuana use, increases crime and raises legal issues, affects public health and safety, and lowers teen educational achievement. Several states have, however, passed regulations that allow the use of marijuana for recreation and/or medical purposes, while some states still maintain that marijuana use is illegal for both recreational and medical purposes. Some states that have not legalized the use of marijuana include Idaho, Wyoming, South Dakota, Nebraska, Kansas, Iowa, Wisconsin, Texas, Indiana, and Kentucky, out of which four states, including Kansas, Nebraska, Idaho, and South Dakota, have completely banned all forms of cannabis and its cannabinoids, including CBD. Although a few years have passed since the legalization of marijuana, it is evident that the green signal to marijuana usage has not yielded much or any benefits to youth and society at large. The statistics provided regarding the impact of marijuana use among people in states that have decriminalized the use of marijuana indicates that although strict rules and regulations have been imposed to prevent the use of marijuana, the number of people using marijuana is on the rise, especially among the youth. Besides, in states where marijuana has been legalized for either medical or recreational reasons, for example, Colorado, Massachusetts, Washington, Maine, and Nevada, higher marijuana usage rates have been reported compared to states such as Texas, Alabama, and Idaho that have legal restrictions. Similarly, in the marijuana-legalized states, marijuana use among youth is also higher than that in states where the ban on marijuana usage is yet to be lifted. Unfortunately, the report on the use of marijuana by teens in the past months has placed marijuana legalization and commercialization pioneers such as the state of Colorado at the top of the list followed by Washington. The increasing number of marijuana-related hospital admissions and marijuana arrests supports this conclusion. At the same time, the percentage of car crashes associated with marijuana use has been increasing although marijuana use and possession are against the law of the states. The worst of it is increased poisoning and consequential hospitalization associated with marijuana and increased rates of road death due to driving while under the influence of the drug. Last, but not least, the existing black market involving Mexican cartels in states like Colorado has grown.

In Colorado, edible marijuana in the form of cakes or gummies accounts for more than half of the marijuana products. These edibles contain THC, a concentrated compound of cannabis. We have not been able to analyze any reports related to edibles. However, more than three deaths in 2015 owing to the consumption of these products confirms the lethality of such edibles. 

The literature analyzed included several poorly designed and contradictive studies. For instance, the Healthy Kids Colorado Survey (HKCS) has contributed to the CDC's Youth Behavior Risk Survey (YBRS). The HKCS data are not truly representative as the second and third most populated counties have been excluded from the survey. Moreover, the study’s statistical significance threshold is significantly higher than expected. Therefore, the results are unreliable due to the flawed methodologies. Currently, the CDC does not use HKCS for YBRS. 

Simultaneously, the initial assurances of crime reduction and tax appropriations have not seen the light of day. The current data about the crime rates associated with marijuana are contrary to the earlier anticipation that legalization would lower the crime rate. Mirroring the case of budget misappropriation or deprivation is the state of Washington, where the share of the promised marijuana tax money has been directed to general funds of the state, contrary to the designed schools and prevention purposes.

When we analyzed the prevalence and criminal rate data of the “marijuana-friendly” states in this study, we found an increase in homelessness. Between the years 2013 and 2014, the national-wide level of homelessness was moving down, courtesy of recession exodus. Unfortunately, though, Colorado was among the 17 states that experienced increased homelessness during the same period (2012-2015). Coincidently, in the same period, recreational marijuana was legalized and commercialization was allowed. The 20% to 30% increase in homelessness was associated with access and proximity to cannabis.

Our review suggested that the commercialization of marijuana is more concentrated among the black and hispanic communities. These communities are reported to endure more disparate consequences of drug usage. Our conclusion concurs with a similar study conducted at John Hopkins, which indicates that low-income black neighbors of Baltimore were at approximately 10 times higher likelihood of being involved in the retail drug business in comparison to other races.

We concluded that the legalization of marijuana has affected the workforce and business in general. According to Quest Diagnostics, 47% of the US workforce tested positive for oral marijuana between 2013 and 2015. More analyses expounded that the rate has swollen to an alarming 178% from 2011 to 2015. However, it is still unclear whether consumption of cannabis worsens the performance of users while increasing the rate of absenteeism. 

Previously, it has been suggested that marijuana legalization would substitute the consumption of alcohol. Contrarily, the intake of alcohol in Colorado has not declined, but has grown, although by a small margin, since the legalization of marijuana.

This brings the question of whether marijuana use should be legalized in the states for recreational and/or medical purposes. Even though the statistical data of the impact of marijuana use are different in the legal and illegal states, there is no wide gap in the values. The above-mentioned detrimental effects of marijuana use can be attributed to both the federal and state governments' failure to execute their duties on the public monitoring and outcome documentation system on eight precise consequences of marijuana use. Disappointingly, there has been no report by the Department of Justice (DOJ) concerning the compliance of the state marijuana program with at least one of the identified criteria.

## Conclusions

In this study, we examined the key factors and relevant outcomes of marijuana pre- and post-legalization, including various effects on society, changes in the public health industry, and medical and psychosocial factors. We compared several states based on the outlined criteria and grouped them into two categories. Attempts were made to determine the consequences of legalization and the related monetary costs through close reading and analysis of various reports of US state and local governments, although the results were contradictory. The evidence provided in this article indicates that strong claims about cannabis legalization, whether by advocates or opponents, are still questionable. The legalization of marijuana for medical and recreational purposes yielded mixed results. We believe that the industry’s influence on policy should be significantly curtailed. We recommend further research efforts and data collection focusing on the mental health effects of marijuana and the cost of treatment for mental health and addiction owing to increased marijuana use.
